# Dietary Omega-3 Fatty Acid Supplementation Reduces Inflammation in Obese Pregnant Women: A Randomized Double-Blind Controlled Clinical Trial

**DOI:** 10.1371/journal.pone.0137309

**Published:** 2015-09-04

**Authors:** Maricela Haghiac, Xiao-hua Yang, Larraine Presley, Shoi Smith, Shirley Dettelback, Judi Minium, Martha A. Belury, Patrick M. Catalano, Sylvie Hauguel-de Mouzon

**Affiliations:** 1 Department of Reproductive Biology, Center for Reproductive Health, MetroHealth Medical Center, Cleveland, Ohio, United States of America; 2 Department of Human Nutrition, College of Education and Human Ecology, Ohio State University, Columbus, Ohio, United States of America; University of Milan, ITALY

## Abstract

**Objective:**

Long-chain omega 3 fatty acids, eicosapentaenoic acid (EPA, 20:5n-3) and docosahexaenoic acid (DHA, 22:6n-3) exert potent anti-inflammatory properties in humans. This study characterized the effects of omega-3 ω-3 fatty acids supplements (ω-3 FA) on the inflammatory status in the placenta and adipose tissue of overweight/obese pregnant women.

**Study Design:**

A randomized, double-masked controlled trial was conducted in overweight/obese pregnant women that were randomly assigned to receive DHA plus EPA (2g/day) or the equivalent of a placebo twice a day from week 10–16 to term. Inflammatory pathways were characterized in: 1) adipose tissue and placenta of treated vs. untreated women; and 2) adipose and trophoblast cells cultured with long chain FAs.

**Results:**

The sum of plasma DHA and EPA increased by 5.8 fold and ω-3 FA/ ω-6 FA ratio was 1.5 in treated vs. untreated women (p< 0.005). Plasma CRP concentrations were reduced (p<0.001). The adipose tissue and placenta of treated women exhibited a significant decrease in TLR4 adipose and placental expression as well as IL6, IL8, and TNFα In vitro, EPA and DHA suppressed the activation of TLR4, IL6, IL8 induced by palmitate in culture of adipose and trophoblast cells.

**Conclusion:**

Supplementation of overweight/obese pregnant women with dietary ω-3 FAs for >25 weeks reduced inflammation in maternal adipose and the placental tissue. TLR4 appears as a central target of the anti-inflammatory effects at the cellular level.

**Trial Registration:**

ClinicalTrials.gov NCT00957476

## Introduction

Chronic low grade or metabolic inflammation is a central condition in the pathogenesis of obesity-induced insulin resistance. Murine models provide evidence to support that adipose tissue is a primary site where activation of adipokines and inflammatory cascades leads to resistance to insulin action. Overexpression of the pro-inflammatory cytokine MCP-1 in adipose tissue induces whole-body insulin resistance [1] whereas inhibiting the expression of MCP-1 or its receptor CCR-2, protects mice from developing high-fat-diet–induced insulin resistance [2]. Mice overexpressing adiponectin are also protected from developing high-fat diet–induced insulin resistance [3]. Adipose tissue functions as a major regulator of fatty acid metabolism due to its high storage capacity for fatty acids as triacylglycerols, i.e. approximately 15–35% of body weight. Eicosapentaenoic acid (EPA) (20:5n3) and docosahexaenoic acid (DHA) (22: 6n-3) are essential ω-3 fatty acids (FAs) that enhance beta-oxidation and up-regulate mitochondrial biogenesis [4]. They are primarily found in cold sea fish and fish oil [5]. EPA and DHA decreased fasting insulin and HOMA-IR in rats and prevented the development of insulin resistance associated with high-fat and high-sucrose feeding in rodents [6]. In humans, long-term fish oil or a combination of EPA/DHA supplementation delayed the progression of metabolic syndrome to type 2 diabetes and reduced insulin resistance in some but not all studies [7, 8]

In human Western diets, ω-6 FAs are in large excess compared to ω-3 FAs, now reaching ratio close to 25:1 rather than the recommended 3:1 [9, 10]. These observations have raised considerable interest to treat these disorders through non-invasive dietary supplementation [11]. Because the biological effects of ω-3 FAs are dependent on the increased concentration of omega-6 in tissues and blood, trying to modulate this ratio by increasing ω-3 FAs, has been the goal of many clinical trials [12]. Increase in maternal fish consumption during pregnancy increases gestation length, and reduces the risk of pregnancy complications [13, 14] although the mechanisms governing these effects remain uncertain. Obese pregnant women develop greater insulin resistance than normal weight women and increased adipose tissue inflammation [15, 16]. Up-regulation of placental inflammatory pathways with elevated release of pro-inflammatory cytokines also contributes to enhance systemic inflammation in obese women [17, 18].

We hypothesized that dietary supplementation with ω-3 FAs during pregnancy will decrease inflammation, through lowering ω-6 concentrations in maternal blood, adipose tissue and the placenta of overweight and obese pregnant women. The cellular mechanisms of ω-3 FAs action were assessed in vitro in primary cultures of adipose and trophoblast cells isolated from tissues of pregnant women.

## Materials and Methods

### Patient recruitment

A double-blind controlled trial was conducted with pregnant women that were assigned to receive oral 800 mg docosahexaenoic acid (DHA, 22:6n-3) and 1200 mg of eicosapentaenoic acid (EPA, 20:5n-3) for a total of 2,000 mg of omega-3 long-chain polyunsaturated fatty acids, divided into 4 capsules, or matching placebo capsules. All subjects were instructed to take 2 capsules twice a day from enrollment (prior to 16 weeks gestation) until delivery. To assess the effect of ω-3 FAs to significantly reduce inflammation and insulin resistance in our pregnant population, with a power of 80% and with a p = 0.05 to detect a 1.5-fold decrease compared to placebo, a sample size of 120 overweight/obese women (60 placebo and 60 ω-3 FAs supplementation) was necessary, assuming a 16.7% dropout rate (50 subjects in each group). This number of patients was expected to be recruited in a 2 year time. The study was approved by the Institutional Review Board of MetroHealth Medical Center/Case Western Reserve University. Prior to participation, all subjects signed a written informed consent form reviewed and discussed with a study coordinator. (National Institute of Public Health in clinicaltrials.gov: NCT00957476). https://clinicaltrials.gov/ct2/show/NCT00957476?term=NCT00957476&rank=1

Subjects were recruited from September 2009 to August 2011 at MetroHealth Medical Center through advertisements, web site postings, and from the hospital’s telephone referral service. Inclusion criteria were a confirmed singleton pregnancy and BMI (wt/ht^2^) ≥ 25 at the first antenatal visit. Gestational age between 8 weeks and 16 weeks based on clinical and ultrasound prior to 20 weeks gestation. Subjects, other than BMI ≥ 25, were generally healthy. Exclusion criteria were evidence of a known fetal anomaly, regular intake of fish oil supplements (defined as greater than 500 mg per week within the last four weeks), daily use of non-steroidal anti-inflammatory agents and pre-existing metabolic disorder such as hypertension, diabetes or hyperthyroidism. Other exclusion criteria were allergy to fish or fish products, gluten intolerance (placebo contains wheat germ oil) or women who are vegetarians and do not eat any fish, planned termination of pregnancy or delivery at another hospital. Known HIV positive, illicit drug or alcohol abuse during current pregnancy were also exclusion criteria. Consenting eligible women received one week’s worth of placebo capsules. Those who either did not return or had taken less than 50 percent of the placebo capsules were not allowed to participate. Women passing the compliance run-in were randomized using a computer generated randomization table (generated by Emiment, the supplier of the ω-3 FA and placebo). Randomization and treatment assignment were carried out by the research coordinators. Study group assignment was not known by study participants, their health care providers, or the research staff. Blister packs were dispensed monthly at routine obstetrical visits at what time compliance and side effects were assessed. There were two visits in the clinical research unit (CRU) at MetroHealth Medical Center for each study participant. Visit one between 8–16 weeks and visit 2 between 34–36 weeks. The two visits consisted in obtaining information height and weight, obtain a sample of maternal blood for plasma cytokine measurements, fasting glucose and insulin measurements. Dietary information have been collected as a form of food frequency questionnaire. Since they have been partially analyzed, we have chosen not to use incomplete information. In the case of elective cesarean section, subcutaneous abdominal adipose tissue (3–5 g) was obtained at the incision site before opening of the fascia. Placenta tissue and cord blood were obtained immediately after double-clamping of the umbilical cord. Adipose and placenta tissue samples were snap-frozen in liquid nitrogen within 5 min of biopsy (3 patients in placebo and 6 patients in ω-3-treatment group).

### Plasma assays

Plasma glucose was assessed by the glucose oxidase method (Yellow Springs, OH). Plasma insulin and insulin concentrations were measured by ELISA (EMD Millipore Corporation, Billerica, MA). Concentrations of IL8 and IL6 in maternal plasma were measured by Quantikine ELISA kits according to the manufacturer instructions (R&D Systems, MN) with the following CV: 0.4–4.7% and CV 0.1–7.2%, respectively. CRP concentration in maternal plasma was determined by using ELISA (Alpha Diagnostics, TX) according to the manufacturer instructions with CV 0.1–7.2%. Data (mean ± SEM) were expressed as delta changes (visit 2- visit1) in placebo vs. ω-3-PUFA group. Total lipids were extracted from maternal plasma, maternal adipose and placenta tissue with 2:1 (v/v) chloroform:methanol and washed with 0.88% KCl [19]. Fatty acid methyl esters were prepared using 5% hydrochloride acid in methanol at 76°C [20]. Analysis of fatty acid methyl esters was completed by gas chromatography with column and conditions as previously described [21]. Retention times were compared to standards (Matreya, LLC, Pleasant Gap, PA, Supelco, Bellefonte, PA, and Nu-Check Prep Inc, Elysian, MN) and fatty acids are reported as percent of total identified.

### Biological specimen collection

To determine the effect of DHA and EPA at the cellular level in both maternal adipose and placenta tissue, previously collected tissue from an independent subset of 16 pregnant women with a singleton pregnancy recruited at term (38–40 weeks) prior to an elective cesarean section was used. This separate study was approved by the Institutional Review Board of MetroHealth Medical Center, Case Western Reserve University. Written informed consent was obtained prior to obtaining blood and adipose tissue. Maternal blood, adipose tissue and placenta were collected as previously described. Maternal pre-gravid body mass index (BMI) and metabolic characteristics were similar to the women consented to double-blind controlled trial and were obtained from the subjects medical records.

### Isolation of stromal adipose cells

Human stromal vascular cells were isolated from subcutaneous adipose tissue collected following C-section. Adipose tissue samples were snap-frozen in liquid nitrogen within 5 min of biopsy or immediately processed for cell isolation. Adipocytes were isolated by digestion of fresh adipose tissue with 1mg/ml collagenase (Worthington Biochemical, Lakewood, NJ) in Hanks buffered solution for 45 min at 37°C. Cells from the stromal adipose fraction (SAF) were pelleted by centrifugation 20 min at 1500 g. The SAF pellet was re-suspended in erythrocyte lysis buffer, centrifuged, suspended in RPMI medium and counted. One aliquot of SVF cells was immediately frozen and the remaining cells were plated at a density of approximate 1.5–1.8 x 10^6^ cells/well in 12 well culture plates (precoated with 1% gelatin) containing RPMI medium containing 10% FCS and 1% penicillin/streptomycin. All the reagents used for SAF cells isolation were endotoxin-free as evaluated by the Limulus Amoebocyte lysate assay (LONZA, Walskerville, MD).

### Isolation of human trophoblast cells

Human trophoblast cells were isolated from obese pregnant women with otherwise uncomplicated pregnancies by sequential trypsin and DNase digestion followed by gradient centrifugation [22]. Cells were plated into 12-well plates at a density of 1.5 x 10^6^ cells/well and cultured overnight in Iscoves’s modified DMEM culture medium supplemented with 10% FBS and 1% penicillin/streptomycin at 37°C under 5% CO_2_.

### Stimulation experiments using fatty acids

For in vitro experiments, the SAF and trophoblast cells were changed to fresh medium after overnight plating followed by incubation under serum-free culture conditions for 24 hours in the presence or absence of fatty acids: C16 saturated fatty acid: palmitic acid (C16:0; 500 μM)(PA), C18 monounsaturated fatty acid: oleic acid (C18:1; 500 μM)(OA) and 100 ng/ml lipopolysaccharide (LPS) (L4391, Sigma Aldrich, St. Louis, MO) ω-3-PUFA: docosahexaenoic acid (C22:6n3; 50 μM)(DHA), and eicosapentaenoic acid (C20:5n3; 50 μM)(EPA). The fatty acids concentrations were within the appropriate non-toxic concentrations ranging within the physiological range. These concentrations were tested on isolated stromal vascular cells and toxicity was excluded by measuring cell viability by trypan blue exclusion and the lactate dehydrogenase activity in the supernatants (using Cytotoxicity Detection kit (Roche Applied Science))(S1 Fig). Fatty acids were dissolved (1M) in ethanol at 70°C for 10 min followed by combination at 1:10 ratio with 2% fatty-acid-free bovine serum albumin. Prior to each treatment, the palmitic acid (100 mM) was incubated for 10 min at 70°C, while oleic acid (100 mM), docosahexaenoic acid (20 mM) and eicosapentaenoic acid (20 mM) were incubated at 45°C for 10min. The formation of fatty acid-albumin complexes is important to reduce the possible cell toxicity of fatty acids. LPS, EPA, DHA and PA were from Sigma. BSA was fatty acid free.

### Gene expression analysis

Total RNA was obtained from isolated SVF cells or isolated trophoblast cells of obese subjects using Trizol reagent (Invitrogen, Carlsbad, CA). Gene expression was monitored by real-time PCR using a Roche thermal cycler (Roche Applied Science, Indianapolis, IN) with Lightcycler Fast-start DNA Sybr Green 1 master mix and primers from Integrated DNA Technologies (Coralville, IA). Specific primers were designed within the 3’ coding region of the genes: toll-like receptor 4 (TLR-4) (NG-011475) forward: 5’-cccaccactcaccagctaat-3’; reverse: 5’-gccctgtggttcagagaaag-3’; interleukin-6 (IL6) (NM_000600) forward: 5’-tacccccaggagaagattcc-3’; reverse: 5’-ttttctgccagtgcctcttt-3’; tumor necrosis factor alpha (TNFα) (NM-000594) forward: 5’-tccttcagacaccctcaacc-3’; reverse: 5’-aggccccagtttgaattctt-3’; interleukin-8 (IL8)(NM_000584) forward: 5’-gtgcagttttgccaaggagt-3’; reverse: 5’-ctctgcacccagttttcctt-3’; β-actin (NM_001101) forward: 5’-ggacttcgagcaagagatgg-3’; reverse: 5’-agcactgtgttggcgtacag-3’. Quantification of relative gene expression normalized for beta-actin was performed by comparative C_T_ method and expressed as fold difference between groups.

### Protein detection by Western Blot

Total protein lysates were prepared by homogenization of cultured trophoblasts 1.5x10^6^ cells / 50 μl buffer (10 mM Tris pH 8, 130 mM NaCl, 1% Triton X-100, 10 mM Sodium fluoride (NaF), 10 mM Sodium phosphate (NaPi), 10 mM Sodium pyrophosphate (NaPPi) with protease inhibitors (Sigma Aldrich, MO) and centrifuged at 13000 rpm for 10 minutes at 4 C°. Protein concentrations were measured with a BCA protein assay kit (Thermo Scientific, IL). 100 μg total protein per well were loaded on 7.5% SDS-running gel (Bio-Rad, CA) and transferred to nitrocellulose filter (Invitrogen, CA). The membrane was blocked with 5% nonfat milk for 1h, incubated with rabbit polyclonal TLR4 (1:200, Santa Cruz, CA) and β-actin (1:2000, Abcam, MA) overnight then secondary antibodies 1:2000 and 1:6000, respectively for 1h. Amersham ECL Plus Western blotting Reagents (GE Healthcare) was used for detection. Densitometric data of autoradiograms were quantified by Image J.

### Statistical analysis

The data were analyzed according to the intention-to-treat principle. Categorical variables were compared with either the Chi Square or Fisher exact test. Continuous variables were compared using a Student t test after testing for normal distribution. The changes over time (visit 1 to visit 2) were analyzed as the differences in measurements at visit 2 minus visit 1 and compared between groups using Student t-test. Statistical analyses were performed using Statview (version 5.01; SAS InstituteCary, NC) statistical package. Data are presented as mean ± standard deviation and significance was set at p<0.05.

## Results

**Fig 1:**
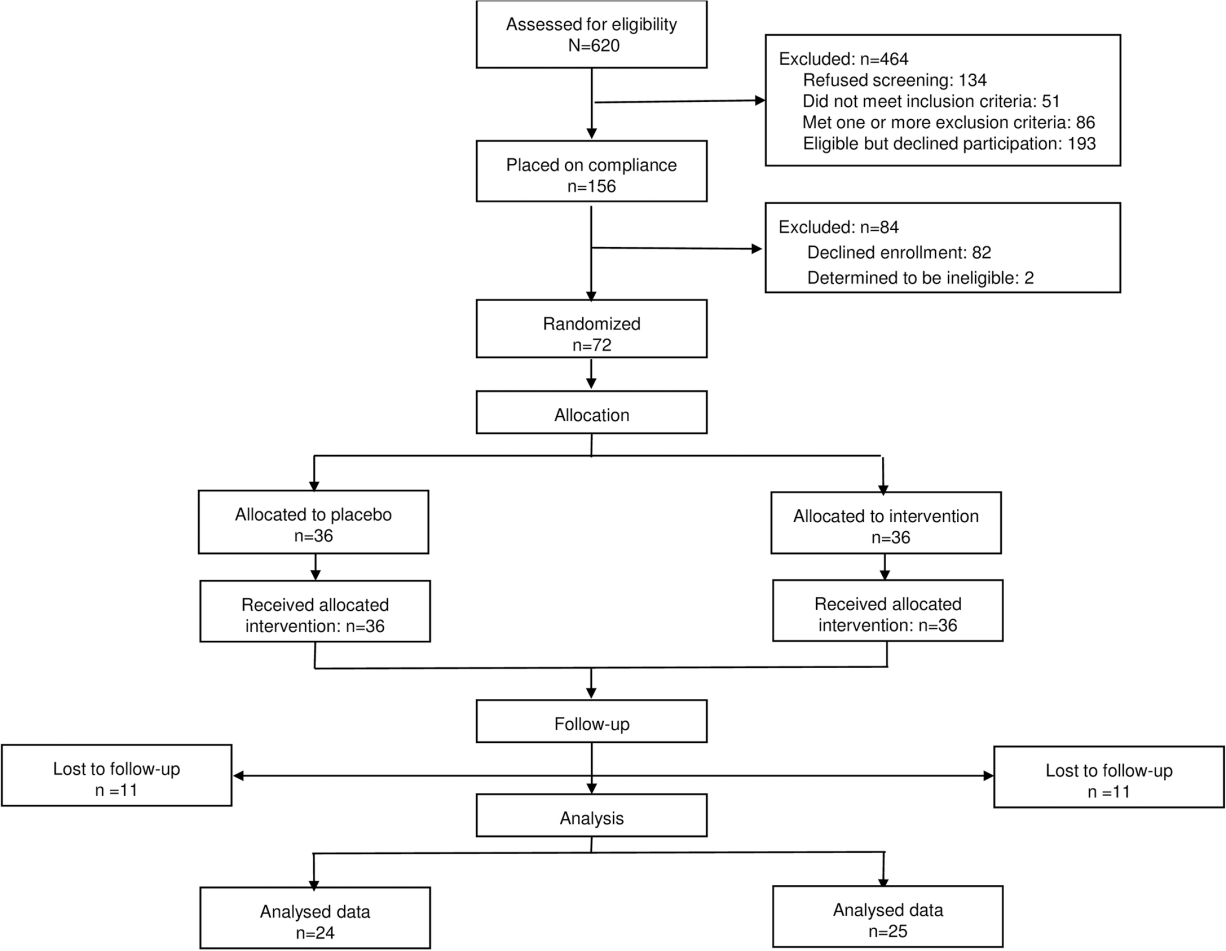
Study Design–Schematic diagram showing how subjects were progressed through the study and how the final study samples were obtained.

The CONSORT diagram is depicted in Fig 1. Six hundred and twenty women were screened, and 156 agreed to be enrolled in the one week run-in. In order to successfully complete the run in the subjects had to take at least 50% of the placebo capsules verified by pill pack count. Eight-four women failed the run in, their age, BMI, ethnicity and parity being similar to the women who completed the run in. Of those who completed the run-in, 36 were randomly assigned to the ω-3 FA supplement arm and 36 to the placebo arm. In the placebo group, 11 were lost to follow up (7 missed visit 2, 1 spontaneous abortion, 2 unable to contact, 1 moved away) and 24 subjects completed the study. In the ω-3 FA group 10 subjects (7 missed visit 2, 1 spontaneous abortion, 1 unable to contact, 1 moved away) were lost to follow-up and 25 subjects completed the study (Fig 1). We only completed studies on 50% of our planned enrollment of 100 subjects. Approximately 1/3 of subjects randomized into the study (21) withdrew before completing the second study visit because of problems taking the 4 capsules/day in early pregnancy. A few subjects had early pregnancy spontaneous abortions documented by ultrasound. One subject in the omega supplementation group developed GDM and had a stillborn at 39 weeks, 2 days after a reactive NST. The autopsy found no cause of death. This was reported to our DSMB and NICHD. There was no significant difference between the anthropometric characteristics of both placebo and omega groups at visit 1 (Table 1). The only significant difference in the ω-3 FA and placebo groups at the time of randomization was a higher body weight (90 ± 20 vs. 80 ± 11kg, p = 0.03) for the women randomized to the ω-3 FA group. Normalization of the parameters by gestational age showed no significant difference between placebo and ω-3 FA groups. At the time of visit 2 the percent EPA (1.05 ± 0.13 vs. 0.18 ± 0.02) and DHA (3.53 ± 0.02 vs. 2.43 ± 0.11) in maternal plasma was significantly increased in the ω-3 FA-treated as compared with the placebo group. As a result the total ω-3 FA and ω-3/ω-6 ratio in maternal plasma at visit 2 was significantly higher in the ω-3 FA treated (p < 0.005) vs. placebo group (Table 2), with no difference between visit 1 and 2 in the placebo group (data not shown).

**Table 1:** Anthropometric characteristics of study cohort at Visit 1.

Subjects	Placebo (24)	ω-3 treated (25)	*p*-value
Maternal Age (years)	27 ± 5	27 ± 5	0.9
GA at randomization (weeks)	14.2± 2	14.3 ± 2	0.2
BMI (kg/m^2^)	32 ± 6	33 ± 6	0.3
Weight (kg)	80 ± 11	90 ± 20	0.03
Weight gain (kg)	8.8 ± 5.1	9.7 ±6.5	0.6
Ethnicity (AA/Cauc/other)	6 / 11 / 7	11 / 10 / 4	0.3
Parity (0 / ≥1)	5 / 19	7 / 18	0.5

Data are means ± SD. *p*-value:comparison between visits within each group. Maternal blood was obtained following fasting. GA-Gestational age; AA-African American; Cauc-Caucasian; other-Hispanic, Asian.

**Table 2:** Maternal fatty acid profile at visit 1 and visit 2.

Fatty acids	Plasma		Adipose Tissue		Placenta tissue	
(% area)	Placebo	ω3-treated	Placebo	ω3-treated	Placebo	ω3-treated
**Eicosapentaenoic acid (EPA)**						
Visit 1	0.25 ± 0.02	0.27 ± 0.03^NS^	N/A	N/A	N/A	N/A
Visit 2	0.18 ± 0.02	1.05 ± 0.13^ɸ^	0.03 ± 0.01	0.03 ± 0.02^NS^	1.9 ± 0.1	1.7 ± 0.1^NS^
**Docosahexaenoic acid (DHA)**						
Visit 1	2.85 ± 0.11	3.03 ± 0.13^NS^	N/A	N/A	N/A	N/A
Visit 2	2.43 ± 0.11	3.53 ± 0.2^ɸ^	0.06 ± 0.01	0.07 ± 0.02^NS^	3.8 ± 2	3.8 ± 1^NS^
**Total ω3 Fatty Acids**						
Visit 1	4.23 ± 0.13	4.41 ± 0.15^NS^	N/A	N/A	N/A	N/A
Visit 2	3.67 ± 0.12	5.82 ± 0.37^ɸ^	1.7 ± 0.3	1.4 ± 0.2^NS^	8.7±1.2	8.2±1.5^NS^
**ω3/ω6 ratio**						
Visit 2	0.09 ± 0.01	0.16 ± 0.01^ɸ^	0.07 ± 0.01	0.07 ± 0.01^NS^	0.5 ±0.04	0.5±0.1^NS^

Data are means ± SD. *p*-value:comparison between placebo and ω-3 treated groups at visit 1 and 2.

ɸ *p* < 0.005

NS-not significant.

**Fig 2:**
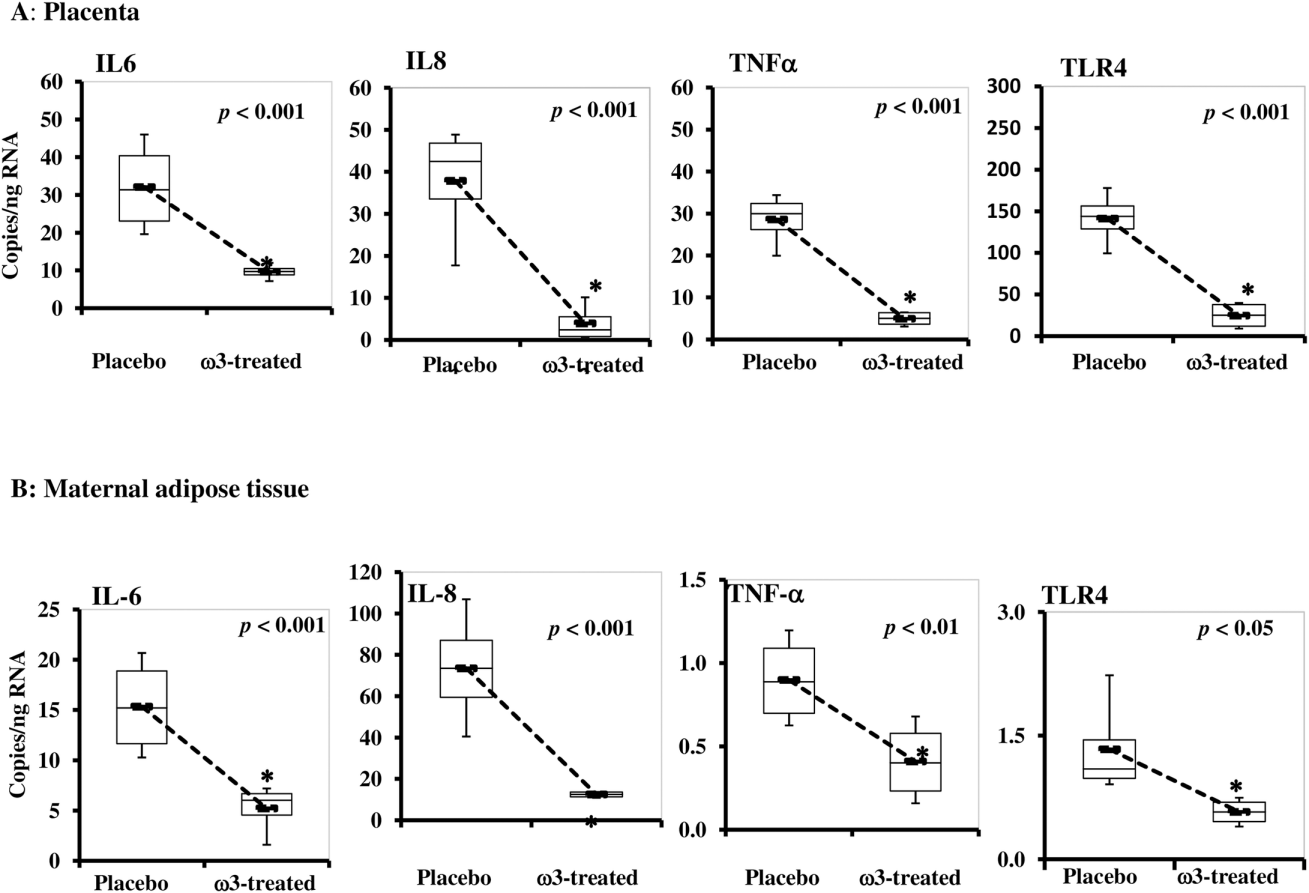
Effect of maternal omega 3 supplementation on inflammatory markers. **A. Placenta.** Quantitative RT-PCR analysis of total RNA isolated from placenta tissue. **B. Maternal white adipose tissue.** Quantitative RT-PCR analysis of total RNA isolated from adipose tissue. Data (mean ± SEM) were expressed as copies per ng RNA in placebo vs. ω3-PUFA treated after normalization to β-actin. Total RNA was isolated from placenta and adipose tissue collected at the time of cesarean section from the recruited women. IL8, IL6, TNFα and TLR4 mRNA levels were measured by quantitative RT-PCR analysis. IL, interleukin; TNFα, tumor necrosis factor alpha; TLR4, toll-like receptor 4; RT-PCR, reverse transcriptase-PCR.

Treated and untreated women has the same delta change HOMA IR index at visit 1 and 2 suggesting that maternal insulin resistance was not modified by omega-3 supplementation. Also there was no difference between the two groups in total ω-3-FAs concentrations in maternal adipose tissue and the placenta (Table 2). There was a significant decrease in the change in maternal plasma CRP from visit 1 to visit 2 in the ω-3 FA group (-3110 ± 5227) vs. placebo group (778 ± 6793) (p< 0.05). There was less of an increase in plasma interleukin 6, but no significant difference in plasma interleukin 8, adiponectin and leptin concentrations between groups (Table 3). The subjects supplemented with ω-3 FA had significantly lower expression of IL6, IL8, TNFα and TLR4 mRNA (p< 0.001) in adipose and placental tissue compared with the placebo group (Fig 2).

**Table 3:** Metabolic parameters of study cohort at Visit 1 and Visit 2.

Subjects	Placebo (24)			ω-3 treated (25)			*p*-value		
	Visit1	Visit2	Δ(V2-V1)	Visit1	Visit2	Δ(V2-V1)	Visit1	Visit2	Δ(V2-V1)
**Glucose (mg/ml)**	80 ± 7	77 ± 6	-3 ± 7	80 ± 6	82 ± 9	2 ± 10	0.9	0.03	0.04
**Insulin (μU/ml)**	6.6 ± 3.0	9.2 ± 5.0	2.6 ± 5.6	9.8 ± 6.7	12.5 ± 6.2	2.7 ± 6	0.06	0.05	0.9
**CRP (μg/ml)**	12.8 ± 9.7	13.6 ± 7.8	0.8 ± 6.9	13.6 ± 11.6	10.4 ± 8.2	-3.2 ± 5.2	0.8	0.2	0.05
**Interleukin 6 (pg/ml)**	1.9 ± 1.5	2.4 ± 1.2	0.5 ± 1.4	2.1 ± 1.5	2.3 ± 1.2	0.1 ± 1.1	0.7	0.6	0.3
**Interleukin 8 (pg/ml)**	2.9 ± 1.2	3.5 ± 1.5	0.5 ± 1.7	3.7 ± 1.5	4.5 ± 1.7	0.6 ± 0.7	0.03	0.02	0.8
**Adiponectin(μg/ml)**	11.7 ± 4.4	9.4 ± 4.4	-2.3 ± 3.7	11.3 ± 4.6	9.7 ± 4.4	-1.9 ± 4.5	0.7	0.8	0.7
**Leptin (ng/ml)**	50 ± 27	54 ± 25	4 ± 20	48 ± 27	58 ± 31	12 ± 23	0.8	0.7	0.3

Data are means ± SD. *p*-value—comparison between placebo and ω-3 treated groups at visit 1, visit 2 and Δ(V2-V1) = Visit2-Visit1. Maternal blood was obtained following fasting.

**Fig 3:**
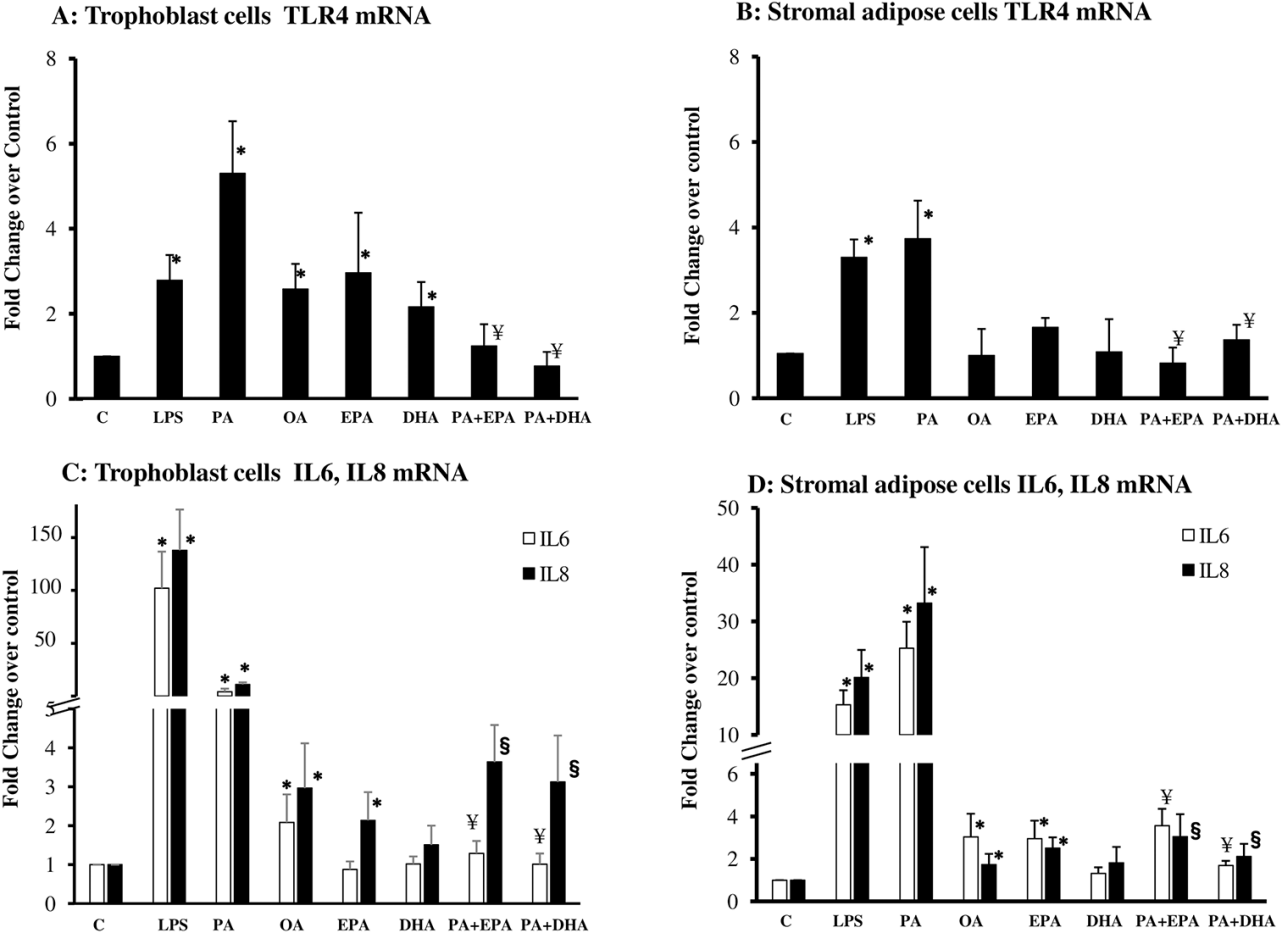
In vitro effects of dietary fatty acids on TLR4 signaling pathways in placental and adipose cells. **A-B. Stimulation of TLR4 mRNA.** Quantitative RT-PCR analysis of TLR 4 from total RNA isolated from cultured trophoblast cells (A) or stromal adipose cells (B)from 4–10 obese women. **C-D. Stimulation of IL6, IL8 mRNA.** Quantitative RT-PCR analysis of IL6 and IL8 from total RNA isolated from cultured trophoblast cells (C) or stromal adipose cells (D)from 4–14 obese women. Cells were stimulated for 24h with 100 ng/ml LPS, PA 500 μM, OA 500 μM, EPA 50 μM and DHA 50 μM. LPS, lipopolysaccharide; PA, palmitic acid; IL, interleukin; TLR4, toll-like receptor 4; OA, oleate; RT-PCR, reverse transcriptase-PCR. Data (mean ± SEM) were expressed as fold changes in FA/ω3-PUFA-treated vs. untreated after normalization to β-actin. Statistical significance: * p< 0.05 vs. control; ^¥^ p< 0.05 vs. PA-stimulation of IL6; ^§^ p< 0.05 vs. PA-stimulation of IL8.

To gain insight into the anti-inflammatory mechanisms of ω-3 FA we conducted in vitro experiments in cells isolated from maternal subcutaneous adipose tissue and placenta. Trophoblast and adipose cells were cultured in the presence of palmitate (PA) and oleate (OA) the two fatty acids most abundant in human adipose tissue [23]. LPS, the natural TLR4 ligand was used as a positive control in both cell types. PA treated trophoblast cells exhibited an increase in TLR4, IL6 and IL8 mRNA expression (Fig 3A and 3C). PA-stimulated cells showed a 5.3-, 8.3- and 10-fold increase (p<0.0001) in TLR4, IL6 and IL8 gene expression respectively, when compared to control untreated cells. OA stimulation of trophoblast cells also increased TLR4, IL6 and IL8 albeit 2–3 times less efficient than PA. EPA by itself induced a modest increase in IL8 and TLR4 but not in IL6. DHA alone was also able to induce a small increase in TLR4 expression (p<0.05). When EPA and DHA were added to the culture medium together with PA, they significantly decreased its inflammatory effect by 66 and 70%, respectively.

The anti-inflammatory effects of EPA and DHA were also analyzed in adipose cells (Fig 3B and 3D). PA induced a 4 to 30 fold increase in TLR4, IL6 and IL8. The stimulation was in the same order of magnitude or even higher than that induced by LPS (Fig 2B and 2D).OA and EPA also induced the expression of IL6 and IL8, although with a 10 times lower magnitude than PA (Fig 3D), while DHA alone did not. Addition of DHA and EPA together with PA decreased the cytokine expression in adipose cells by 61 to 68% for IL8 and by 76 to 80% for IL6. The same inhibitory trend was observed when EPA and DHA were added 2h prior to PA treatment (data not shown). Similar to mRNA, protein data show a decreased expression of TLR4 in trophoblasts in the presence of EPA or DHA (S2 Fig).

These data suggest that addition of DHA or EPA to the culture medium prevented the PA induced-TLR4 and inflammatory cytokines in both adipose and trophoblast cells (Fig 3).

## Discussion

The physiological state of low grade inflammation is enhanced in pregnancy of obese women [16, 17]. The main findings of this study was that ω-3 supplementation in pregnancy resulted in decreased inflammation in overweight/obese women. Following 25 weeks oral supplementation, there was a lower expression of inflammatory genes in adipose tissue and the placenta as well as decreased plasma CRP at the time of delivery. The anti-inflammatory properties of ω-3 FAs are well characterized in obesity [24]. To our knowledge this is the first report showing that ω-3 fatty acid supplementation decreases obesity-associated tissue inflammation in pregnancy. Besides obesity and the metabolic syndrome, ω-3 FAs have protective effects in several conditions associated with chronic inflammation, rheumatoid arthritis, coronary heart disease, and Crohn’s disease [11, 25]. The anti-inflammatory actions of ω-3 FAs are based on their ability to decrease the production of pro-inflammatory cytokines and eicosanoids by several tissues and cells. Diets that are rich in ω-6 fatty acids produce eicosanoids, whereas a diet with a more balanced intake of ω-6 and ω-3 fatty acids makes less inflammatory and less immunosuppressive eicosanoids [7]. While change in dietary intake in essentials fatty acids have immediate consequences on systemic concentrations, accumulation in tissues tends to take longer time ranging from weeks to months. Omega-3 fatty acids are incorporated in tissues with different efficiencies, possibly because of inter-conversions or different affinities of the enzymatic pathways involved [26]. We believe that in this study accumulation of ω-3 FAs in adipose tissue and placenta happened at a rate too slow to raise concentrations during the length of the RCT. EPA and DHA also provide substrates for synthesis of the pro-inflammatory lipid mediators protectins, resolvins and decrease adipokines through inhibition of NF-*K*B signaling [27–31]. Similarly to LPS, PA activates a TLR4-induced inflammatory cascade in trophoblast and adipose cells [32]. All obese individuals do not have increased circulating fatty acids but rather display increased FAs storage in adipose and ectopic tissues such as muscle and liver. This is also the case in obese pregnant women whose fasting plasma FA are generally not elevated compared to lean pregnant women [33]. Palmitate is the most abundant saturated fatty acid constituent of human adipose tissue triglycerides [34] and there is a progressive increase in the proportion of palmitate in maternal blood from first to third trimester [35]. We demonstrated that IL6 and IL8 were the most abundant cytokines released from adipose and trophoblast cells upon palmitate stimulation. Rather than directly lowering cytokine expression, EPA and DHA inhibited the release of palmitate-induced inflammatory cascade. DHA and EPA are essential FA readily transferred from mother to fetus because they are not synthesized by fetal tissues, our findings suggests that there is no regulation of placental storage but rather a systemic effect from either the fetal or the maternal circulating DHA and EPA. These mechanisms of action are in line with data in human endothelial cells [36]. Other studies have reported that treatment of obese humans or rodents with ω-3 FAs led to reduced circulating levels of pro-inflammatory cytokines and acute phase proteins [37, 38]. Of note, the decreased expression of the inflammatory cytokines in adipose tissue was not associated with a significant decrease in cytokine concentrations in the plasma of the treated women. Although adipose tissue is an important source of IL6 it has been estimated to account for only 30% of IL6 in the organism [39]. Inflammatory cytokines have essentially a local impact at the cellular level primarily paracrine in adipose tissue between adipocytes and SVF and autocrine in trophoblast cells [40, 41]. Furthermore, the release ability of cytokines varies with anatomical origin of the cells and the type of cytokine [42]. Hence cytokines synthesized in tissues and cells are more likely to be released locally rather than in the systemic circulation. For this reason we did not expect that the anti-inflammatory effects observed at tissue level would translate into similar decrease in plasma cytokine concentrations. Additionally, obesity is a chronic LOW grade inflammation [17, 43] with low circulating levels of inflammatory cytokines so that it is unlikely that an ELISA assay is sensitive enough to detect modest decrease in cytokine plasma concentration.

Other studies have reported that ω-3 supplementation during pregnancy does not prevent recurrent preterm birth in asymptomatic singleton gestations with prior preterm birth [44]. However there was an increased birth weight in the treated group. Whether the discrepancies between studies may be related to differences in study design, dosage or length of treatment, additional RCTs are needed to clarify the impact of ω-3 on these outcomes. Collectively, the data from the current study and those in non-pregnant individuals suggest that several tissue or cell types such as Kupffer cells, endothelial cells, monocytes may also contribute to regulate plasma IL6. In the current trial, the best marker for improved systemic inflammation was plasma maternal CRP concentration with a 6-fold decrease between ω-3 treated and placebo groups. Because IL6 is a major regulator of CRP production [45] it is conceivable that down-regulation of IL6 in adipose tissue may have initiated the decreased CRP secretion.

The strength of this clinical trial was the significant anti-inflammatory effect of omega-3 supplementation in placenta and adipose tissue of obese pregnant women. However, we noticed a high dropout rate, partially related to the discomfort associated with taking 4 capsules/day. This resulted in a smaller than expected number of subjects enrolled in the trial. Because of the metabolic and inflammatory changes taking place at very early stages of pregnancy [46] it may prove an event more efficient strategy to supplement obese women before they get pregnant.

In conclusion, our results provide support for our hypothesis that ω-3 FAs decrease inflammation in obese pregnant women through decreasing the TLR4-induced innate immune response in adipose and trophoblast cells. These findings encourage further studies to define the precise TLR4 pathways elicited by omega-3 FAs in the anti-inflammatory cascade.

## Supporting Information

S1 CONSORT Checklist CONSORT 2010 Checklist of information to include when reporting a randomized trial. (DOC)

S1 Fig Effect of PA and OA treatments on viability of cultured stromal adipose cells. Quantitative RT-PCR analysis of IL6, IL8 and TNFα. Total RNA was isolated from cultured stromal adipose cells stimulated with different PA concentrations from at least 10 obese women. *p< 0.05. B. Cell viability measured by trypan blue exclusion in isolated stromal adipose cells treated with increasing concentrations of PA and OA (n = 3). C. Lactate dehydrogenase activity in supernatants following treatment with different concentrations of PA and OA (n = 10). PA, palmitic acid, OA, oleate. *p< 0.05.
(TIFF)

S2 Fig Effect of FA stimulation on TLR4 protein expression in trophoblast cells. Cultured trophoblast cells treated for 24h with 100 ng/ml LPS, 500 μM myristic acid (MA), palmitic acid (PA), stearic acid (SA), oleic acid (OA), linoleic acid (LA), 50 μM EPA and 50 μM DHA or in the absence of fatty acids (CTL). A: Representative Western Blot B: densitometry analysis of n = 3 independent experiments.
(TIFF)

S1 Protocol Detailed protocol IRB for the omega-3 supplementation study in pregnancy (trial NCT00957476). (PDF)
