# Supplementary figures and images for: Dietary Omega-3 Fatty Acid Supplementation Reduces Inflammation in Obese Pregnant Women: A Randomized Double-Blind Controlled Clinical Trial
Source: PLoS One. 2015 Sep 4;10(9):e0137309. doi: 10.1371/journal.pone.0137309 (PMC4560373; doi:10.1371/journal.pone.0137309)

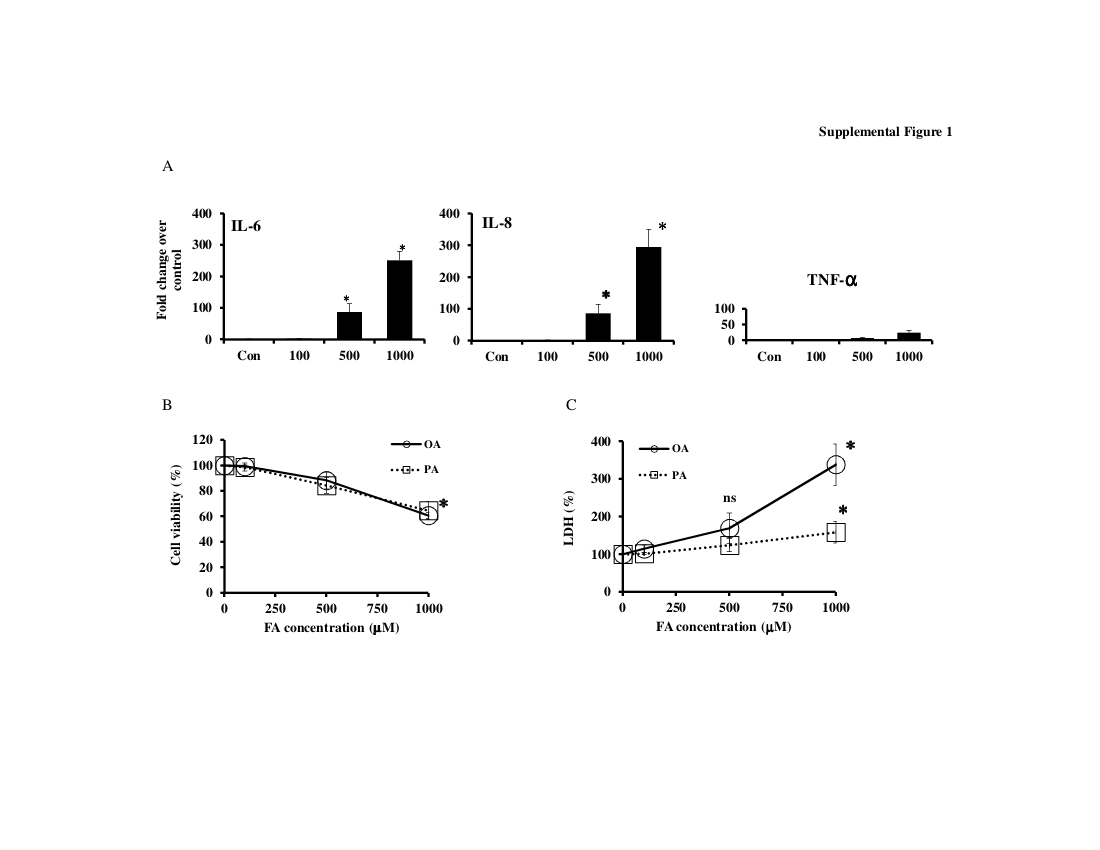

Supplement: S1 Fig — Quantitative RT-PCR analysis of IL6, IL8 and TNFα. Total RNA was isolated from cultured stromal adipose cells stimulated with different PA concentrations from at least 10 obese women. *p< 0.05. B. Cell viability measured by trypan blue exclusion in isolated stromal adipose cells treated with increasing concentrations of PA and OA (n = 3). C. Lactate dehydrogenase activity in supernatants following treatment with different concentrations of PA and OA (n = 10). PA, palmitic acid, OA, oleate. *p< 0.05. (TIFF) [file pone.0137309.s002.tiff]

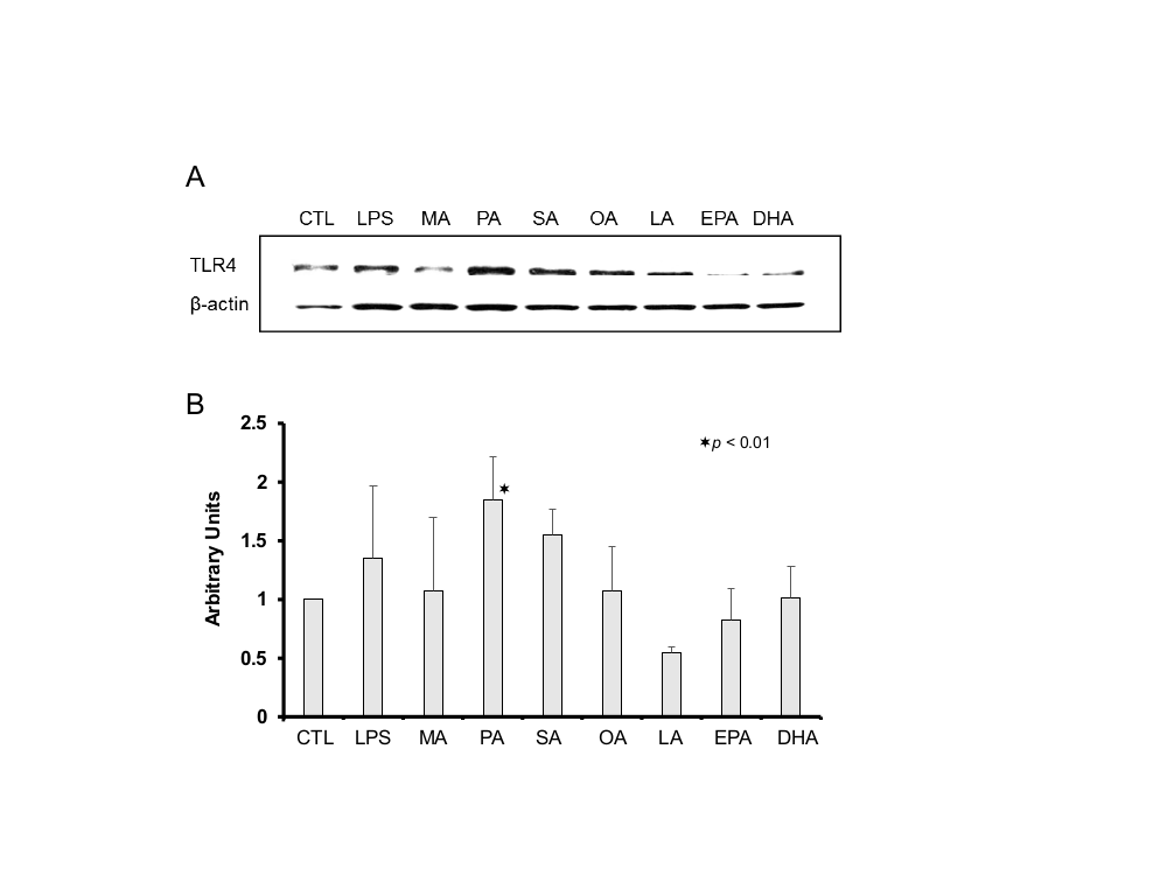

Supplement: S2 Fig — Cultured trophoblast cells treated for 24h with 100 ng/ml LPS, 500 μM myristic acid (MA), palmitic acid (PA), stearic acid (SA), oleic acid (OA), linoleic acid (LA), 50 μM EPA and 50 μM DHA or in the absence of fatty acids (CTL). A: Representative Western Blot B: densitometry analysis of n = 3 independent experiments. (TIFF) [file pone.0137309.s003.tiff]
