# Supplementary material for: Dietary Omega-3 Fatty Acid Supplementation Reduces Inflammation in Obese Pregnant Women: A Randomized Double-Blind Controlled Clinical Trial
Source: PLoS One. 2015 Sep 4;10(9):e0137309. doi: 10.1371/journal.pone.0137309 (PMC4560373; doi:10.1371/journal.pone.0137309)
Supplement: S1 Protocol — (PDF) [file pone.0137309.s004.pdf]

MetroHealth

Date: Wednesday, February 11, 2015 10:16:02 AM
View: 01-00 Study Information

Print | Close

1.0 Study Information:

1.1

* **Short Title:** Omega-3 Supplementation in Pregnancy

The Short Title Should be the sponsor protocol number. If there is no sponsor protocol, then enter 3-5 words or numbers that capture the important study characteristics and help identify the study.

1.2

* **Full Title of Research Project:**
Omega-3 fatty acid supplementation during pregnancy improves insulin sensitivity and decreases inflammation in overweight/obese women.

Enter the Full Title of the study.

1.3

Principal Investigator: Patrick Catalano

HSR Certification Status: Certified **HSR Certification Expiration Date:** 9/1/2016 ;

COI Expire Date: 8/20/2015 ; **COI Yes or No:** No ; **COI Management Plan:** ; **PI Non-Compliance:**

The PI must be a MetroHealth Staff person or have privileges to practice at MHS. The PI must assume full responsibility for the conduct of the study.

1.4

Key Personnel:

	Name	CREC Status	CREC Expiration	COI	COI Expire	Management Plan?	Study Roles	Employer Name	Non-Compliance
View	Praneeta Chodavarapu	Certified	7/27/2016	no	10/8/2015		Research Support Staff	Case Western Reserve University	
View	Perrie O'Tierney-Ginn	Certified	3/6/2016	no	4/29/2015		Research Support Staff	The MetroHealth System	
View	Sylvie Hauguel-de Mouzon	Certified	4/6/2017	no	6/20/2015		Interviewer (Survey, Focus Group) Obtaining Informed Consent Co-investigator	The MetroHealth System	
View	Shoi Smith	Certified	9/11/2015	no	7/9/2015		Research Support Staff Interviewer (Survey, Focus Group) Obtaining Informed Consent	Obstetrics/Gynecology	
View	Sharon Groh -Wargo, PhD	Certified	9/1/2016	yes	10/15/2015	yes	Interviewer (Survey, Focus Group) Obtaining Informed Consent Co-investigator	The MetroHealth System	
View	Stephen Myers	Certified	1/23/2016	no	9/13/2015		Research Support Staff	The MetroHealth System	
View	Larraine Presley	Certified	9/1/2016	no	8/20/2015		Research Support	Obstetrics/Gynecology	

Add additional Staff as needed.

Update to add Study Roles

	Name	CREC Status	CREC Expiration	COI	COI Expire	Management Plan?	Study Roles	Employer Name	Non-Compliance
							Staff Interviewer (Survey, Focus Group) Study Coordinator Obtaining Informed Consent DRA (only one)		
View	Patrick Catalano	Certified	9/1/2016	no	8/20/2015		Interviewer (Survey, Focus Group) Obtaining Informed Consent	The MetroHealth System	

1.5 Type of Research:
Physiologic Research

1.6 If "Other" Type of Research Please Explain:

View: 01-01 Study Information

1.1 Study Information:

1.7 * Department-What Department approvals are required?

Name
Obstetrics/Gynecology

1.9

Definitions to keep in mind when selecting the degree of risk:

<u>Minimal Risk</u> is defined in 45CFR46 and in FDA regulations 21CFR50.3 as:
Minimal risk means that the probability and magnitude of harm or discomfort anticipated in the research are not greater in and of themselves than those ordinarily encountered in daily life or during the performance of routine physical or psychological examinations or tests.

*** Degree of Risk: (This is the investigator's assessment of the risks involved in the research which will informed the IRB Decision but which will not automatically be accepted. The Board is the final arbiter of risk. The risk level will be set by the IRB staff at the time of approval.)**

Select most appropriate one.

	Name
○	Risk
●	**Not Greater Than Minimal Risk**

1.10 * Type of IRB Review Requested:
Full Board

Select one. If you select Exempt or Expedited you will be taken to that section when you hit continue.

View: 01-02 Study Information

1.2 Study Information:

1.11 **Will you require access to Epic to conduct this study?** (•) **Yes** ( ) No

The DRA's employee number must be listed on their registration form.

If you answer this question yes you will need to identify a Designated Records Administrator one person only.

Please add DRA to roles on Page 1 Key personnel

1.12 **Is the Principal Investigator a resident or trainee?**
( ) Yes (•) **No**

Please check yes or no.

If the answer to 1.11 is yes, then you must have a MHS Staff member who assumes full responsibility for the conduct of the study. This individual along with all other personnel involved in the study, must be current with their Human Subject Research training requirements. This role should be given to one perosn in the Key personnel on the first page of the study.

Please add Desingnated Responsible Investigator to roles on Page 1 Key personnel

View: 01-03 Study Information

1.3 Study Information:

1.13
*** Will CRU Be Used:**
Yes *If you answer yes to this question this application will be sent to the CRU for review after departmental review and before it is submitted to the IRB.*

Will the CRU be used?

1.14
*** Has this research protocol ever been submitted to another CASE affiliated IRB (i.e. UH, CCF, VA or CASE)?**
No

If this study has been reviewed at another CASE affiliated IRB you should answer yes.

1.15
If yes, was it:

Select one from drop down menu.

1.16 **Please supply the following information: At which institution was it approved? If it was disapproved, why was it disapproved?**

What institutions have approved this study. If it has been disapproved, please give a brief explanation of why study was disapproved.

Please attach the Approval letter/letters from other IRBs (i.e. UH, CCF, VA or CASE):

Name	Description
There are no items to display	

1.17

Please attach approval letter/letters.

View: CRU 01-01 Application

Please Note: If you are using the CRU you must adhere to the following New NIH Public Access Policy:

Please review the information provied by theis link regarding enforcement of the NIH Public Access Policy that will begin on April 1, 2013. The most recent changes to the NIH Public Policy are explained in the attached Power point presentation from the NIH (January 15, 2013) and the attached MS-Word document, "Manuscript Submission to PubMed Central for a PMCID".

All studies that utilize the MetroHealth CRU resources (space, nursing, lab, bionutrition) that are non-industry funded, are required be NIH Public Access Policy compliant by obtaining a PMCID number. The PMCID number is an separate index from the PMID - the PMCID number indexes the entire publication while the PMID indexes the abstract, only. In addition to the PMCID number, investigators are required to post their publications on 'My Bibliography' and link their publications to grant numbers.

Attention to this policy is important because the NIH will halt the process of renewals, re-submissions and certain progress reports if relevant publications are non-compliant with the PMCID number and My Bibliography. Continued use and funding of the CRU may be jeopardized if appropriate publications are not fully compliant.

In addition, please ensure that studies utilizing the CRU also acknowledge the CTSC grant in their publications and cite the CTSC grant number, **UL1TR000439**. This is a NEW NIH CTSC grant number that went into effect on June 1, 2012. The acknowledgment and grant number can also be found by going to the Cleveland CTSC website acknowledgments page. (This page also has information about the NIH Public Access Policy.)

Thank you in advance for ensuring that all publications from studies utilizing CRU resources (that are not-industry supported) are compliant with these requirements as soon as possible.

1.00 CRU Application [*Since you have indicated in your application that you want to utilize the resources of the CRU Please complete the following pages of the IRB Application. Hit the continue button to move from page to page in that way you will be able to take advantage of the built in branching logic to complete your CRU application.*]

1.01 **Is this an HIV/AIDS Project:** ○ Yes ◉ **No**

HIV/AIDS?

1.02 **Are you currently Funded by NIH?** ◉ **Yes** ○ No

If yes please add grant or contract number.

1.03 **Would you like to conduct your study on the CRU?**
◉ **Yes** ○ No

Yes or No

1.04 **What is your eRA Commons Name?**
pcatalano

eRA Commons Name required for all non- industry studies

1.05 **Anticipated Start Date:**
9/1/2009

Anticipated Date Study to Begin (1st Patient)

Anticipated End Date:
9/3/2012

Anticipated End Date

1.06 **Approximate Inpatient Days Per Subject:**
0

Enter approximate numbers

Approximate Outpatient Visits Per Subject:
2

1.07 *After August 2010 this is a read only copy of the paper CRU Resource Application:*

Old CRU Application forms

Name	Version
There are no items to display	

1.08 **These are your target enrollment numbers for the ethic and racial categories below:**

Enter anticipated numbers for this protocol

Ethnic Category

	Sex/Gender		
Ethnic Category	Females	Males	**Total**
Hispanic or Latino	20	0	20
Not Hispanic or Latino	100	0	100
Ethnic Category Total of All Subjects	120	0	120

Racial Category

	Females	Males	**Total**
American Indian/Alaska Native	0	0	0
Asian	2	0	2
Native Hawaiian or Other Pacific Islander	0	0	0

Black or African American	85	0	85
White	33	0	33
Racial Categories Total of all Subjects	120	0	120

View: CRU 01-02 CRU Resource Needs

2.00 CRU: Resource Needs

Hospital Lab Tests: (•) **Yes** ( ) No

Hospital Lab Tests = CRU draws blood and send it to Hospital Lab or forwards blood already drawn to Hospital Lab

Human Performance Lab Tests and Measurements: (•) **Yes** ( ) No

Human Performance Lab Tests and Measurements

Research Nutrition Services: ( ) Yes (•) **No**

Research Nutrition Services

CRU Laboratory Services: (•) **Yes** ( ) No

Core Laboratory Services (7:00 am to 5:30 pm, weekdays)

Use of CRU Facilities or Equipment Only: (•) **Yes** ( ) No

Use of CRU Faciities or Equipment Only

Nursing Resources: (•) **Yes** ( ) No

Use CRU Nurses

Spanish Translation Services: ( ) Yes (•) **No**

Translation is the rendering of a written text in one language in a comparable written text in another language

Spanish Interpertation Services: ( ) Yes (•) **No**

Interpreting is the oral rendering of spoken or signed communication from one language into another.

View: CRU 01-03 CRU Nursing Resources

1.03 CRU: Nursing Resources

3.01 **CRU Nursing Visits:**

Please Add Each Visit

	Title	Visit #	Visit Length	Admissions	Comments
View	CRU Visits #1 and #2	2	4 Hours	no	

View: CRU 01-04 Core Laboratory Services

4.00 CRU: Laboratory Services

4.01
Sample Processing: ◉ **Yes** ○ No

4.02 **DNA Isolation:** ○ Yes ◉ **No**

4.03
Packing for Shipping: ○ Yes ◉ **No**

4.04
Other Lab Procedures:
CBC on 1st visit only. All others are batched.

4.05
Instances of Shipping and Processing:
No specimens are shipped.

View: CRU 01-05 Hospital Lab Tests

1.05 CRU: Hospital Lab Tests

Required for research purposes. Include Standard of Care test to be drawn and sent to the MHMC central lab here.

5.01 **CRU Hospital Lab Tests:**

Please give the details of any Lab tests needed.

	EPIC Order Code	Hospital Lab Test Name	Number Of Tests Per Subject
View	700001409	CBC	1

View: CRU 01-06 Human Performance Lab Tests and Measurements

1.06 CRU: Human Performance Lab Tests and Measurementes

6.01
Exercise Training: ○ Yes ◉ **No**

Answer Yes or No

6.02 **Exercise Training Description:**

Describe the exercise training

How many times per week

6.03
Exercise Training # Times/Week:

Answer Yes or No

6.04
Treadmill: ○ Yes ◉ **No**

6.05
Treadmill Description:

Description of treadmill exercises

6.06 **Ergocycle:** ○ Yes ◉ **No**

Answer Yes or No

6.07 **Ergocycle Description:**

Describe the use of the ergocycle

6.08 **Other Activities:** ○ Yes ◉ **No**

Answer Yes or No

6.09 **Description of Other Activities:**

Describe "Other" activities

View: CRU 01-08 Use of CRU Facilities or Equipment Only

8.00 CRU: Use of CRU Facilities or Equipment Only

8.01
Use Of Equipemnt: ○ Yes ◉ **No**

8.02 **Use Of Equipment List:**

8.03
Use Of Space: (•) **Yes** ( ) No

8.04
Use Of Space Description:
Metabolic Lab for OGTT

8.05
Other (Specify):

View: CRU 01-09 Additional Notes or Requests

1.09 CRU: Additional Notes or Requests

9.01
If you have any additional notes or requests from the Clinical Research Unit that have not been covered, please describe them here:

View: 01-04 Study Information

1.4 Study Information

These Questions are specifically about the adequacy of resources, are there the necessary resources to complete this study? There are two questions which focus on nursing resources. If this research will require the use of nursing resources then the Nursing Resources Form found on the IRB Home Page under forms and templates will need to be completed and attached to this research application.

1.18 Can you assure the IRB that there are adequate numbers of qualified staff to conduct this research?
(•) **Yes** ( ) No

Please answer yes or no. This is an assurance to the IRB.

1.19 How will the investigator ensure that persons assisting with the research were adequately informed about the protocol and their research-related duties and functions and requirements for maintaining the confidentiality of all data?
The study staff has been working for the PI many years and is CREC certified by MHMC IRB. The study staff is well-versed in the HIPAA policies as well as all procedures and requirements for maininting confidentiality during and after data colection. The study staff will be trained on all protocol-related procedures prior to the start-up of the study.

i.e. investigator meeting, formal protocol review with PI, monitor, sponsor.

1.20 Will the PI and study staff have sufficient time to conduct and complete the research?
(•) **Yes** ( ) No

Please answer yes or no. This is an assurance to the IRB.

1.21
What facilities are available to conduct the research? Are they adequate? Please describe.
The study will eventually be performed in the CRU. The facilities in the CRU are more than adequate. The PI has been performing similar studies in the CRU for the past 20 years.

Please describe the facilities, i.e. lab, procedure room, chemo treatment room.

Nursing Resources:

1.22
Is this study using MetroHealth staff nurse time or labor ? (i.e. giving medcations, teaching, or additional documentation)

( ) Yes (•) **No**

This is in addition to the time of the study/research nurse.

1.23 Attach Nursing Resources Form here:

Click here for Nursing Resources form

Open the form, Complete the form and save it to your files then attach it to the study by hitting the browse file and selecting the file and hitting OK.

Click here for the MHS Policy

View: 04-00 Scientific Review

4.0 Scientific Review:

All Studies need a Science Review. Has your study been reviewed by any of the following?

4.1 Please Check all that Appy to this study so that the IRB may make a determination if there needs to be further scientific review:

Select all that apply. Note FDA Approval does not equal science review.

Review Type

- [ ] Initiated and sponsored by industry under an IND, IDE, HDE, or 510K exemption issued by the FDA for which no scientific integrity concerns were identified during the FDA review process
- [ ] Trial initiated and sponsored by industry that has undergone a scientific merit review by the sponsoring agency, but is not being conducted under an IND, IDE, HDE, or 510K exemption
- [ ] Sponsored by a Cooperative Group
- [ ] Proposed research has been awarded funding by a federal agency
- [x] **Peer reviewed by a federal funding agency and received a favorable funding score**
- [ ] Peer reviewed by a federal funding agency with the acknowledgment of scientific merits, but not likely to be funded for reasons unrelated to scientific merit
- [ ] Sponsored by a foundation or a private agency that requires a separate scientific merit review process at the sponsoring agency
- [ ] No Science Review

4.2 Do any of the following apply to your study? Please check all that apply:

Additional Reasons Why Science Review May Be Required

Investigator-initiated study

Check all that apply your answers will assist the IRB in deciding if further science review is necessary.

4.3 Does this study require review by the Biosafety Committee?

No

All studies involving vaccines, potentially hazardous materials or genetic research must go to the biosafety committee at CASE.

4.4 Does this study require review by the Radiation safety committee? No

If a study involves more than routine exposure to radiation on the part of subjects the study must go to the radiation safety committee.

4.5 Does this study require Review by the Nursing Committee?

No

The nursing committee must review all studies where the PI is a nurse, and all studies which have as the primary objective to contribute to nursing knowledgebase, and/or have implications for nursing practice.

View: 05-00 Funding Information I

5.0 Funding Information I:

All Research Projects must have an identified funding source!

5.1 Is this research externally funded? Yes

Check one

Research can be both externally and internally funded so you can answer yes to both 5.1 and 5.6.

5.2 Types Of External Funding:

Name

Government / Federal

Check all that apply.

5.3 If other, external funding please explain:

If other please describe.

5.4 Sponsor Information:

Name Sponsor/Agency	Address	Telephone	FAX	Contact Person
NICHD, NIH - Pending	6100 Executive Blvd., Room 4B03, MSC 7510	Tel. 301-496-5577		Caroline Signore, MD, MPH

Please supply this information as your application can not be processed without it.

5.5

Have you received and/or submitted a Notice of Award or Contract?
No

Select one from drop down menu.

Attach notice of award.

If yes, attach your Notice of Award letter here (not your grant):

Name Version
There are no items to display

View: 05-01 Funding Information II

5.1 Funding Information II

5.6

Is Research Internally Funded (internal funding is any MetroHealth System or MetroHealth Foundation funds): ○ *Yes* ◉ ***No***

Check one, research can be both externally and internally funded so you can answer yes to both 5.1 and 5.6.

5.7 Internal Funding Sources List:
Internal Funding Source
There are no items to display

Check all the apply.

5.8

If a MetroHealth Foundation funds or any MetroHealth System funds are being used, has department approval been received?
○ Yes ○ No

Check yes or no.

5.9

If a MetroHealth Foundation funds or any MetroHealth System funds are being used indicate the Account Number:

Please enter the account number if this applies.

5.10

*** Are there current Conflict of Interest Forms for all Key Personnel? [It is the responsibility of the Principal Investigator to ascertain this information and check this box.]**
◉ **Yes** ○ No

This question is not asking if there are COI forms for all Key Personnel it is asking if all Key Personnel have current COI forms so that any SFI is reported and can be dealt with if a management plan is need or reporting to NIH is required.

In order to submit a new protocol all COI Forms for key personnel and investigators must be current = provide up to date information.

5.11 Please check below any Conflicts of Interest (Financial) you as Principal Investigator or your study staff [Co-Investigator, Coordinators, Other Study Staff] may have on this Study:
Potential Conflict of Interest
None of the above options apply and there are no other financial conflicts of interest in the conduct of this research.

This question pertains to this study and is not a general question. Check all that apply.

You and/or your study staff will need to file a Conflict of Interest Disclosure Form annually.

If anyone working on this study has a Conflict of Interest or a perceived conflict. This information will need to be included in the consent form i.e. company is paying MHS to do this study.

5.12 Please attach a copy of your grant application here:

Name	Description
Revised Grant Application	

You must attach a copy of your grant application here (i.e. NIH Grant Application).

You have the option to attach a copy of the budget, clinical trial account authorization form, contract and Approval letter(s) now or you can email them to your grants management specialist in the RABO office.

Copies of all RABO forms are available at:

http://www.metrohealthresearch.org/raboforms.html

View: 06-00 Performance Site Information

6.0 Performance Site Information:

6.1
At what sites will the study team be performing this research, (please enter information about all non-MHS sites in 5.2):

Select all that apply. If you select other please enter information about that site in question 6.2.

Name
The MetroHealth System

If this study is being done at MetroHealth where is it being done give the physical location (i.e. 8B, ED, Broadway, Old Brooklyn, PICU, Cath Lab):
CRU

Where is the research going to be done? What physical location on the Main campus or the community health centers?

6.2 Please provide information about other external sites here:

Name of Site	Address	Telephone Number

There are no items to display

Please enter contact information. Please include name of facility, address and department.

6.3 If you are doing this research at an external site does this site have an IRB?
○ Yes ○ No

Select yes or no.

6.4 If the External Site has an IRB will that IRB defer review to the MHS IRB?
○ Yes ○ No

This only applies if there is no IRB or if there is a legal agreement between institutions permitting a reciprocal review, i.e. CASE.

6.5 Attach letter from external site agreeing to permit the MHS to review this protocol:

Name	Description

There are no items to display

Attach letter.

6.6 Has the external site granted permission for the research to be conducted?
○ Yes ○ No

This applies to sites where there is no IRB and the investigator must get a letter from the site that gives permission to conduct the research at the site.

6.7 Attach letter from external site granting permission for the research to be conducted:

Name	Description

There are no items to display

Attach letter of support.

View: 06-01 Performance Site Information

6.1 Performance Site Information

6.8
Is MHS the lead institution of a multi-site study? ○ Yes ◉ **No**

Please answer yes or no.

6.9 If yes, is there a plan to communicate information obtained through research that might be relevant to the protection of human subjects, including a plan to provide the IRB with information on unanticipated events, interim results, and protocol modifications.
○ **Yes** ○ **No**

Please answer yes or no.

6.10 Please give a detailed explanation of the above plan:

This plan must give the IRB enough information to decide if the plan is appropriate and adequate.

6.11 Will the Principal Investigator conduct this study at any location outside the United States of America?
Yes **No** (selected)

Answer these questions only if there are research sites outside the USA.

6.12 Country, City, and address:
Country Address of Research Facility
There are no items to display

Give country and location.

View: 07-00 Research Objectives and Background

7.0 Research Objectives and Background:

7.1 * ABSTRACT: Please give the IRB a 500 word Abstract that contains the specific objectives of the study.

This is your abstract also known as a synopsis from an industry sponsored study. Please limit to 500 words.

In addition to the increase in obesity in adult and children, there has been a significant increase in birth weights over the last 2 decades. Based on our preliminary data, maternal pre-gravid obesity is the strongest risk factor for neonatal as well as adolescent obesity. The long-term goals of our research are to examine therapeutic strategies to decrease fetal adiposity. Obesity and pregnancy are both insulin resistant conditions associated with chronic low-grade inflammation. Therefore, we hypothesize that n-3 PUFA dietary supplements during pregnancy will act as insulin sensitizers decreasing peripheral insulin resistance and inflammation. If correct this mechanism should decrease availability of maternal nutrients to the fetus and subsequently reduce adiposity at birth. We plan a prospective randomized double blind control trial of n-3 PUFA supplementation and placebo in overweight/obese women, with a planned cesarean delivery, initiated in early pregnancy and maintained throughout pregnancy. This proposal has two specific aims.
Specific aim 1 is to evaluate the effect of n-3 PUFA supplementation on maternal insulin sensitivity. Measures of maternal insulin sensitivity and lipid metabolism will be made using the ISogtt, indirect calorimetry (base on availability of staff), body composition (BODPOD) and plasma lipid profile at baseline and after dietary intervention.
Specific aim 2 will assess the effect of n-3 PUFA on the inflammatory status in overweight/obese pregnant women. We hypothesize that n-3 PUFA supplementation decreases chronic inflammation during pregnancy by preventing monocyte activation and accumulation of macrophages in WAT thus lowering systemic concentration of pro-inflammatory cytokines. We plan to characterize the longitudinal changes in circulating monocytes and plasma adipokines in order to define the inflammatory patterns in both groups over time. We will also determine the abundance and phenotype of macrophages infiltrating WAT using flow cytometry, immunohistochemistry and gene expression profiling. Furthermore, the role of PPAR-gamma as a central target of n-3 PUFA action to regulate insulin sensitivity will be examined by characterizing the expression of PPAR-gamma in WAT of both supplemented and control groups. Additionally, we will investigate the direct affect of n-3 PUFA on the expression of adiponectin and PPAR-gamma regulated genes in primary cultured adipocytes.
In summary, this proposal combines both clinical and molecular methodologies in an overweight/obese subject population in order to assess the effect of n-3 PUFA on inflammation and insulin resistance. Preliminary data will also be obtained on fetal body composition in order to later address the prevention of the long term adverse effects (developmental programming) of maternal obesity in the developing fetus.

7.2 * What are the specific aims of this study i.e. what are the question(s) this research intends to answer? Provide at a maximum 3 primary and 3 secondary aims.

This is your Hypothesis also know as your aims (NIH) or safety and efficacy aims (industry). Please list no more than 3 primary and 3 secondary clearly label these aims primary and secondary.

The long term goals of our research have been to understand the affect of maternal metabolism on feto-placental growth, in particular neonatal body composition and the long term effects on the offspring. In this proposal our overall hypothesis is that the abnormal metabolic milieu of maternal obesity is instrumental for in utero programming of excess fetal adipose tissue deposition.

In this amended application we will focus our efforts primarily on the second specific aim of the original proposal based on the 2 year granting period of the Recovery Act or ARRA. We will assess: 1) the effect of n-3 PUFAs on maternal insulin sensitivity and inflammatory markers and 2) the molecular mechanisms by which n-3 PUFAs modify maternal white adipose tissue (WAT) inflammation. In contrast to the original application we will not have the power to assess the effect of n-3 PUFAs on fetal adiposity or enough resources to evaluate the effects of n-3 PUFAs on placental inflammatory gene expression.
Obesity and pregnancy are both insulin resistant conditions characterized by chronic low-grade inflammation, as manifested by increased plasma concentrations of cytokines, acute phase reactants and activation of inflammatory signals. We hypothesize that n-3 PUFA supplementation during gestation will decrease the inflammatory response in maternal WAT. Subsequently, decreased maternal insulin resistance, resulting from the lower level of inflammation, will contribute to limit nutrient availability to the fetus thereby resulting in lesser neonatal adiposity.

Specific aim 1:
To evaluate the effect of n-3 PUFA supplementation on maternal insulin

resistance and inflammatory markers in overweight/obese women.
This specific aim will test the hypothesis that n-3 PUFA dietary supplementation begun in early gestation will significantly decrease maternal insulin sensitivity and inflammation. We postulate that n-3 PUFA supplementation in overweight/obese women will modify the concentration of circulating cytokines resulting in a decrease in maternal insulin sensitivity . This then results in decreased lipid and glucose availability for maternal-fetal placental transport and resultant fat accretion in the fetus, to be more fully examined in future studies. We will test our hypothesis by:

1. Conducting a prospective randomized double blind control trial of n-3 PUFA supplementation in women with an early pregnancy BMI of > 25, estimating maternal insulin sensitivity and measures of lipid metabolism in the basal and insulin stimulated conditions using ISogtt, body composition and indirect calorimetry, at baseline and after dietary intervention.

2. Examining the effect of n-3 PUFA supplementation on maternal fatty acid and lipid profiles at baseline and after supplementation.

Specific aim 2:
To assess the molecular mechanisms of n-3 PUFA action on adipose tissue inflammatory responses
Pregnancy is a physiologic condition of chronic low-grade inflammation which is further amplified by obesity. In obese pregnant women, there is an increased expression of macrophages and macrophage secreted factors in circulating peripheral white blood cells. Consequently, macrophages accumulate in maternal WAT resulting in a higher production of pro-inflammatory factors. We postulate that giving an n-3 PUFA enriched diet to obese pregnant women will decrease accumulation of macrophages and related factors in their WAT thus reducing the chronic inflammatory environment in which the fetus develops. We will test our hypothesis by:
1. Evaluating the systemic inflammatory profile in n-3 PUFA supplemented and placebo treated women. The peripheral mononuclear blood cells (PMNC) will be counted to estimate leucocytosis in early and late pregnancy. The expression of IL-6, TNF-alpha (pro-inflammatory cytokines), CCR-2 and IL8-R (chemoattractant protein receptors) will be measured by realtime RT-PCR of CD14+ PMNC to characterize their activation state. Plasma concentrations of maternal pro-inflammatory cytokines, adiponectin and acute phase reactants will be also measured by ELISA.
2. Characterizing the inflammatory profile in WAT by analysis of the resident macrophage populations (number, phenotype and secretome) in n-3 PUFA supplemented and control groups.
3. Additionally, we postulate that activation of the transcription factor PPAR-gamma in adipose tissue plays a central role in the mechanisms of n-3 PUFA action to reduce global insulin resistance. We will test our hypothesis by measuring the expression of PPAR-gamma gene and its target adiponectin in adipose tissue as well as adiponectin plasma concentration in n-3 PUFA supplemented and control groups. We also will investigate the role of PPAR-gamma as a direct target of n-3 PUFA action in cultured adipocytes and adipose tissue macrophages isolated from subcutaneous WAT of obese pregnant women undergoing term elective cesarean section.

7.3 Please provide a summary of the present knowledge relevant to the research and make citation to any applicable scientific literature:

This is your literature search and bibliography. Also known as Background and significance (NIH) or Introductory Section from industry sponsored trial.

BACKGROUND AND SIGNIFICANCE

1) Obesity and Pregnancy: the Propagation of a Vicious Cycle
In women of reproductive age in the United States, the prevalence of obesity defined as body mass index (BMI >30) was 30.2% while the prevalence of overweight (BMI>25) was 56.7% in the latest CDC reports. The prevalence of obesity in adolescents has also increased by 11.3% between 1994 and 2000. In Denmark the percentage of macrosomic newborns (birth weight > 4 kg) increased from 16.7% in 1990 to 20.0% in 1999. In Cleveland, mean birth weight at term has increased 116 g over the past 25 years with higher maternal weight having the strongest correlation with the increase in birth weight. We also recently reported that the neonates of overweight/obese women have increased fat but not lean body mass as compared with infants of non-obese women. Save for bariatric surgery, most forms of obesity treatment yield only short term results. Hence, prevention of obesity rather than treatment is preferable. However, when should prevention begin? Based on our data, the cycle of obesity begins in utero, with increased birth weight, because of increased adiposity.

2) Fetal Programming/Fetal Origins Hypothesis
The process of fetal programming has received considerable attention as a potential etiology for the observed increase in the prevalence of obesity and metabolic syndrome. Whitaker recently reported that among low-income children, maternal obesity in early pregnancy doubles the risk of obesity at 2-4 years of age. Boney et al also reported that although LGA offspring of gestational diabetic (GDM) mothers were at increased risk for the development of the metabolic syndrome in childhood, children exposed to prenatal maternal obesity alone were also at risk, suggesting that obese women have factors independent of GDM affecting postnatal outcomes. This is not surprising given that the criteria for GDM were based on the maternal risks for the development of type-2 diabetes and not fetal macrosomia. We therefore hypothesize that treatment of obese mothers

ideally before but at least in early pregnancy may be the initial step in the prevention of these disorders in later life. Maternal pregravid obesity is not only a risk factor for neonatal obesity but has the strongest correlation with childhood obesity and the metabolic syndrome.

3) Fetal Growth
We have focused on neonatal body composition as a more specific measure of the processes affecting fetal growth, as genetic factors have a stronger relationship to lean body mass, while fetal fat accretion related to the in utero metabolic environment. The average fetal weight at the end of the second trimester is 1000 g, therefore 70% of fetal growth including the vast majority of fetal fat accretion, accrues during the third trimester of gestation. Not surprisingly, the majority of studies evaluating fetal growth have concentrated on metabolism in late pregnancy. The combination of high insulin and NEFAs, associated with increased maternal insulin resistance supported by the broader concept of "fuel mediated teratogenesis" has long been regarded as the primary determinant of fetal fat accumulation. However, decreased maternal pregravid insulin sensitivity has the strongest correlation with adiposity at birth and, increased pregravid maternal BMI, a marker of decreased insulin sensitivity, also is positively correlated with adiposity at birth. These data are consistent with the epidemiologic studies of Whittaker and Boney describing increased maternal pregravid BMI as a significant risk factor for obesity and metabolic syndrome in the offspring of obese women.
How then to ameliorate the decreases in insulin sensitivity during pregnancy in obese women? Life style measures of diet and exercise in order to decrease weight prior to a planned pregnancy are the ideal, but not realistic given that few pregnancies are planned. Insulin sensitizers such as metformin or thiazolidinediones are theoretically useful, but they both cross the placenta and safety has not been documented. Low glycemic index diets may be useful to improve insulin resistance and response, but the data on efficacy are controversial at best. Total caloric restriction may be the better dietary approach to decrease weight gain, but during pregnancy when the obese women are already at risk for "accelerated starvation", the association between gestational ketonemia in the mother and lower IQ scores in the child at 4 years of age warrants concern regarding adverse outcomes with hypocaloric diets. Weight-bearing exercise starting at 8 weeks gestation such as on a treadmill for at least 40 minutes 5 times/week at an intensity 55-60% of preconception maximum aerobic capacity through the remainder of gestation, resulted in infants with lower body fat (8.3% vs. 12.1%, $p<0.01$) as compared with women who decreased exercise by 24 weeks gestation. The amenability of obese women to such a rigorous exercise regimen and the ability to continue throughout pregnancy is guarded at best. In summary, there are few realistic safe options available for obese women to limit the decrease in insulin resistance and effects on lipid metabolism in pregnancy.

4) n-3 Polyunsaturated Fatty Acids
Increased intake of n-3 PUFAs docosahexaenoic acid (DHA) and eicosapentaenoic acid (EPA) decreases fat cell mass, improve insulin sensitivity through decreased NEFA and triglyceride concentrations. In rodents, n-3 PUFAs have a protective effect against high fat diet induced insulin resistance by preventing alterations of insulin signaling in muscle, liver and adipose tissue. At this time, the mechanisms by which n-3 PUFAs affect insulin sensitivity and lipid metabolism in human are speculative but supplementation clearly decreases plasma triglycerides, leptin gene expression and protein content in white adipose tissue (WAT) and placenta through regulation of PPARgamma. Shulman's group recently published data suggesting that fish oil is a regulator of adiponectin secretion in vivo through PPAR-gamma dependent gene induction in epididymal fat.
During pregnancy, dietary supplementation with n-3 PUFAs has been promoted as a means to prolong gestation and the NICHD conducted a prospective randomized trial to test this hypothesis. In the older n-3 PUFA trials, there was a purported increase in birth weight in the supplemented women related to increased gestational age, however, recent studies adjusting for gestational age report a decrease in birth weight. Similarly, Groh-Wargo, a Co-Investigator in this proposal, reported decreased fat, but not lean body mass in infants at 1 year of age whose formulas were fortified with n-3 PUFAs. Although the USA expert panel recommends that pregnant women consume at least 300 mg/day of DHA (31), the mean intake of DHA for pregnant and lactating women was only 52 mg/day and 20 mg/day for EPA. This may in part be explained by the decrease in fish consumption, after the FDA issued an advisory counseling to pregnant women to avoid consuming fish which may contain high levels of mercury. Because n-3 PUFAs are essential fatty acids, they can only be obtained through the diet as alpha-linoleic acid and converted to EPA and DHA, though only at a rate of between 1-4%. With n-3 PUFA supplementation, there is a positive relationship between maternal intake and maternal plasma concentration as there is between maternal plasma and cord concentrations. In the human, there is an inverse correlation between cord DHA and birth weight. We hypothesize that by supplementing obese women with n-3 PUFAs, we will be able to decrease the rate of maternal lipolysis and thus decrease transplacental NEFA availability for fetal fat accretion.

7.4 Option to Upload Documents related to question 7.3:

If it is easier to attach your response to question 7.3 please do so here. Please limit to three pages.

Name	Description
There are no items to display	

View: 08-00 Methods and Procedures I

8.0 Methods and Procedures I:

8.1 Will this research involve the following Social-Behavioral Procedures:

Check all that apply.

Name
Surveys/Questionnaires

8.2 Will this research involved any of the following Medical Procedures/Considerations:

Check all that apply.

Name
Study of Human Biological Materials (i.e. Urine Collection)
Study of Existing Data
Medical Tests, Comparisons, Evaluations
Clinical Assessments (EEG, EKG, SCID, etc.)
Venipuncture (Blood Draw)
Anthropomorphic Measurments

8.3 Identify Data Collection types for this study:

Check all that apply.

Note if you are doing, recordings, Video-Recording/Photographs then subjects will need to sign the MetroHealth Audio-Video Consent form. See the IRB Forms and Templates.

Name
Banking of Specimens/Data
Chart Review - Prospective
Existing/Retrospective Data/Specimens
Interviews, questionnaires or psychological tests
Anthropomorphic evaluations

View: 08-01 Methods and Procedures II

8.1 Methods and Procedures II:

8.4 * Please specify in detail the methods and procedures that are involved in this research:

If this field is not completed your protocol will not be reviewed. Do not enter N/A. Please describe what methods and procedures will be involved in this research.

Clinical evaluation of patients
Each subject will be admitted to the CRU. At enrollment subjects will have routine laboratory studies. The assessment of activity level in study subjects will be assessed using the Minnesota leisure time activity questionnaire. The nutritional data obtained from subjects includes total caloric intake, the percent carbohydrates, fat and protein as well as specific measures of n-3 PUFA intake using a validated Harvard School of Public Health food frequency questionnaire designed specifically to assess n-3 fatty acids. Dietary guidelines for all subjects are those currently recommended by the Institute of Medicine for obese pregnant women. Subjects will be instructed in the use of the food frequency questionnaire by the nutritionist at the start of the run in period, one week prior to the first outpatient day study in the CRU. A 75 g 2 hour oral glucose tolerance test (OGTT) will be used to define normal glucose tolerance and GDM using the criteria of the Fourth International Work Shop Conference on Gestational Diabetes. Insulin sensitivity will be estimated during the 75 g OGTT by obtaining 2 baseline venous samples for glucose and insulin at 15, 30, 45, 60, 90 and 120 minutes. Insulin sensitivity (ISogtt) will be calculated as described. This methodology has been tested in pregnant women in comparison with euglycemic clamps by our group and correlates well (r2 =0.74, p=0.0001). Plasma NEFA concentrations will be measured at baseline and at 15, 30, 45, 60, 90 and 120 minutes during insulin response to the 75 g glucose challenge to measure suppression of lipolysis.

The same testing will occur at 34-36 weeks gestation after each subject has been randomized to n-3 PUFA or placebo with the exception of the blood which will not include the CBC, HgA1C, Creatinine, Calcium and Phosphorous and TSH. All testing will remain the same.

Anthropometric measures and placental morphometrics
Maternal body composition will be measured in early and late pregnancy using air displacement technology (Bod Pod). The Bod Pod was compared with both hydrodensitometry and X-ray absorptiometry (DEXA) and compares to within 1% of body fat for adults. We plan to use previously published equations to estimate body composition in pregnant subjects accounting for the variation in hydration of lean body mass in pregnancy. The within subject and day to day coefficient of variation for the percent body fat using Bod Pod was between 2-3% comparable to other methods. Neonatal body composition will be obtained within 7 days of birth, if possible. Neonatal body composition will be measured using a pediatric air displacement methodology (PEA POD). The PEA POD uses the same principle of air displacement to measure body density in infants between birth (2 kg) and 6 months of age. The device has been shown to accurately measure weights and volumes as compared with standards of the National Institute of Standards and Technology. The coefficient of variation in volume measurements is < 0.05%. In a measure of animal carcass phantoms with a percent body fat of 18%, the Pea Pod correlated percent body fat was r2 =0.99 and the 95% CI was + 1.1%. Last in comparison with a 4 compartment model (deuterium dilution, DEXA, total body potassium and mass) and PEA POD, there was no significant difference in percent body fat (17.7 + 5.4 vs. 18.1 + 6.7%, p=0.60) between the 2 methods. We plan to utilize this measure in addition to anthropometry to estimate neonatal adiposity in newborns of our study subjects. Distribution of body fat and anthropometric estimates of body composition will be assessed using 5 skinfold measures (triceps, subscapular, umbilical, flank and thigh).
Placental weight will be recorded at delivery. The umbilical cord will be cut at the insertion site in the placenta and membranes removed before weighing. Placental biopsies obtained only at the time of elective cesarean sections within 10 min of delivery, snap frozen in liquid nitrogen and stored at −80°C. Additionally, 2 mm width tissue slices with full placenta thickness (chorionic to basal plate) will be excised and fixed in 4 % paraformaldehyde prior to paraffin embedding. Venous cord blood will be obtained at delivery by puncture of the clamped umbilical cord, only on scheduled cesarean sections.
Placenta assays (specific to cesarean sections): Lipid profile will include concentration of

saturated fatty acids (14:0-24:0), monounsaturated (14:1, 16:1, 18:1, 20:1, 22:1), as well as n-3, n-6 and n-9 PUFAs and their eicosanoid products. Frozen plasma and placenta aliquots will be used for lipid extraction. Measurements will be performed in the phospholipids, neutral and total lipid fractions. All phospholipids and neutral lipids are separated on Silicagel 60, saponified and methylated. Fatty acid methyl esters are separated and quantified on a Perkin-Elmer gas chromatograph. Spots are quantified as integrated optical densities (IOD) against an internal standard of cholesteryl formate.
In the group of women undergoing cesarean delivery, placenta and subcutaneous WAT biopsies will be used for analysis of tissue macrophages. Analysis will be performed by indirect immunodetection methods (immunofluorescence of tissue section and flow cytometry of isolated cells) and measurement of gene expression after isolation and purification of tissue macrophages. Once we have obtained a sufficient number of samples for macrophages analysis, the WAT samples from the placebo group will only be used to isolate adipocytes for in vitro experiments. For this specific aim of the study, the subjects will be unblinded immediately after delivery to ensure we are only using adipose tissue from the placebo group. Based on our power analysis 10-20 cell culture experiments from individual subjects should be sufficient to reach statistical significance in the characterization of PPAR-gamma dependent mechanisms of n-3 PUFAs.

The efficiency of n-3 PUFA to reduce the overall inflammation state will be assessed through :
1. Characterization of tissue macrophages by indirect immunofluorescence of paraffin sections of WAT and placenta using the macrophage markers CD68 and CD14 and assessment of lipid content.
2. Assessment of the effects of n-3 PUFA supplementation on the secretory phenotype of resident macrophages. The gene expression profile will be obtained by microarray analysis of the adipose tissue and placental macrophages (Affymetrix oligonucleotide microarrays U133) from the supplemented and placebo groups.
3. Assessment of the effects of n-3 PUFA supplementation on reducing systemic inflammation and activation of PMNC.
4. Evaluation of systemic inflammation by characterizing the activation state of maternal PMNC at delivery: monocytes and granulocytes count (flow cytometry), expression (real time PCR) of macrophages markers (CD68, CD14, EMR1), chemokines receptors (CCR-2 and IL-8R), chemokines (MCP-1, Il-8) and pro-inflammatory cytokines (TNF-alpha, IL-1, IL-6). The systemic inflammation will also be assessed by measuring plasma cytokines (TNF-alpha, IL-6, IL-1, adiponectin) and acute phase reactants (serum amyloid A, serum amyloid protein, C-reactive protein) in maternal and cord plasma in placebo and supplemented groups.

The efficiency of n-3 PUFA to activate adiponectin through PPAR-gamma dependent mechanisms will be assessed in adipose tissue and isolated adipose cells as follow:
1. The expression of PPAR-gamma and its target adiponectin will be measured by real time RT-PCR in WAT of supplemented and placebo treated women.
2. The ability of EPA and DHA to stimulate PPAR-gamma and adiponectin gene expression and transcriptional activity will be evaluated in cultures of adipocytes isolated from WAT of obese pregnant women. The effect of PPAR-gamma to enhance adiponectin production will be assessed by inhibiting PPAR-gamma activity using specific PPAR-gamma inhibitors (bisphenol-A -diglycidyl ether, PPAR-gamma siRNAs
3. Measures of adiponectin released in culture medium of adipocytes before and after incubation with EPA and DHA.
4. Measures of lipid insulin sensitivity (insulin-mediated inhibition of lipolysis) before and after incubation with EPA and DHA
5. The effect of EPA and DHA on PPAR-gamma regulated pathways will also be directly evaluated on macrophages isolated from WAT of obese pregnant women.

Rationale for Specific Aim #2 (WAT biopsy):
Tissue resident macrophages are the primary cell population responsible for propagating immune and inflammatory responses at the cellular level. Our preliminary data show that obesity during pregnancy is associated with an increased number of macrophages in adipose tissue and placenta. The increase in tissue macrophages in obese pregnant women is associated with an activation of maternal PMNC and higher plasma cytokines concentration which are signs of systemic inflammation.
A primary beneficial effect of n-3 PUFA enriched diet is through a reduction of macrophage accumulation in WAT. We hypothesize that n-3 PUFA will decrease inflammation not only in WAT but also in the placenta of n-3 PUFA supplemented women by preventing in situ accumulation of macrophages. We will test our hypothesis by:
1) Identifying the resident macrophage populations (abundance, surface markers) in women treated by n-3 PUFA and placebo.
2) Characterizing the phenotype of the macrophage subsets and their state of activation in women treated by n-3 PUFA and placebo. Additionally, we will investigate whether n-3 PUFA decrease systemic inflammation by measuring gene expression of inflammatory mediators in maternal PMNC. The combined secretory capacity of PMNC and tissue resident macrophages will be assessed by measuring concentrations of inflammatory cytokines in plasma of n-3 PUFA and placebo supplemented women.
A potential mechanism of n-3 PUFAs action is the regulation of genes involved in adipogenesis via the transcriptional activity of the peroxisome proliferator activated receptors PPAR-gamma. Activation of PPAR-gamma is associated with increased adiponectin and adiponectin receptor gene expression suggesting a functional link between PPAR-gamma and adiponectin signaling. Our preliminary data indicate that PPAR-gamma and adiponectin gene expression are decreased in WAT of obese pregnant women. We postulate that activation of PPAR-gamma in adipose tissue will restore adiponectin levels and thus ameliorate some of the decrease in insulin sensitivity. We will test our hypothesis by measuring PPAR-gamma and adiponectin gene expression in WAT and plasma adiponectin in n-3 PUFA supplemented and control groups. Secondarily, we will investigate whether PPAR-gamma is a primary cellular target of n-3 PUFA

action in adipocytes and adipose tissue macrophages. Using adipocytes and isolated macrophages we will assess the effect of EPA and DHA to increase adiponectin production.

For those who cannot tolerate having the OGTT done, for whatever reason, then a fasting glucose test will be substituted without changing the outcome of the study.

8.5 Does this study only involve the use of existing/retrospective data/specimens?
No

Check yes or no.

8.6 Describe in <u>detail</u> the study design also known as the experimental flow. Include all study procedures a subject will go through, in order of sequence and timing, including frequency of visits, duration of visits, length of subject participation etc. ***Please Note this needs to be written for an educated person who is not an expert in the field, do not exceed 300 words*:**

This is also known as NIH Experimental Procedure section or Clinical Trial Procedure/Experimental Flow section. Do not just attach documents in response to this question you must do a study design summary for IRB Review.

Subjects will be recruited in the antenatal clinic of MHMC by the clinical research specialist. Maternal height and weight will be obtained from the patient's antenatal chart to verify BMI criteria are met. All subjects eligible for the study will undergo a "run in period" of 1 week to ensure that they consume at least 50% of placebo capsules. We use 50% or greater as the criteria for a successful run n period because 8 capsules (the dose used in the NICHD clinical trial to prevent premature delivery at MHMC) are required to ensure intake of 800 mg DHA and 1200 mg EPA. Those successfully completing the run in will be randomized in blocks of 10, to either placebo or n-3 PUFA supplement.
After successfully completing the run in period, gestational age will be confirmed as appropriate, for example clinical exam, beta HCG, early ultrasound, etc. Subjects will have their initial metabolic studies in the CRU on or before 16 completed week's gestation. Each patient's history and physical exam will be reviewed from their antenatal chart to ensure that the she is in good health. During this time the subject's height and weight will be verified to ensure that each subject meets study guidelines. Prior to the metabolic studies in the CRU, the subject may eat up until 22:00 the previous evening and remain fasted, with the exception of water, until the following morning. At or around 0800 the patient will have laboratory studies including a complete blood count, hemoglobin A1c, TSH, creatinine, calcium and phosphorus, lipid profile (cholesterol & triglyceride), additional lipid studies (e.g. Omega-3 NEFA, etc.), and cytokines such as CRP, TNF-alpha, etc. A 2 hour 75 g ISogtt will be performed to estimate: 1) glucose tolerance, 2) insulin sensitivity, 3) NEFA and glycerol concentrations in response to insulin, and 4) fat oxidation using indirect calorimetry(based on staff availability)and 5) UUN-post OGTT. During this visit subjects will also have a food frequency questionnaire under the supervision of the nutritionist to estimate not only total calories and percent of protein, carbohydrates and fat but also specific intake of n-3 PUFA. The assessment of activity level in study subjects will be assessed using the Minnesota leisure time activity questionnaire. After completing the ISogtt each subject will have a measure of body composition. At the conclusion of the studies each subject will be randomized to receive either placebo or n-3 PUFA supplementation. We have estimated the ratio of EPA and DHA based on the content in fish for e.g. Atlantic mackerel, salmon and herring, lake trout, etc to be 0.66. Of note this is the ratio of EPA to DHA used in the NICHD trial to prevent preterm delivery. The total daily dose will be 800 mg of DHA and 1200 mg EPA. The formulation of the soft gel capsules has proved to be indistinguishable from the placebo (mineral oil and wheat germ oil) without a telltale "fishy taste". Safety issues of n-3 PUFA are discussed in the human subjects section. The subjects will return to the CRU after each scheduled clinic visit to bring back their pill packs to account for compliance and to receive new pill packs. The subjects will return to the CRU between 34 to 36 weeks to repeat the protocol to determine the changes over time and between groups.
At delivery, placental weight will be recorded. We will obtain venous cord blood for measures of glucose, C-peptide, NEFA, n-3 PUFAs and cytokines (based on staff availability). Within 7 days of birth, neonatal body composition will be measured using the Pea Pod. Distribution of body fat and anthropometric estimates of body composition will be assessed using 5 skinfold measures (triceps, subscapular, umbilical, flank and thigh). All of this contingent on when and if, the mother or parents are able to bring in the newborn.
To standardize data collection and minimize variability in placental gene expression due to difference in labor management and maternal metabolic status at delivery, when available. The rate of cesarean delivery in obese women who have had a previous cesarean section is approximately 70% at MHMC so that we expect to analyze between 20-30 placentas in each group. Placental tissue will be processed in Dr. Hauguel-de Mouzon's laboratory in the Rammelkamp research building. Tissue samples will be immediately snap-frozen in liquid nitrogen and stored at -80 until RNA extraction. Additional fragments will be fixed in 4 % paraformaldehyde prior to paraffin section for immunohistochemistry. The placenta from placebo and n-3 PUFA study groups (approx 20-30 in each group) will have focused gene array studies to assess for changes in genes related to lipid metabolism, cytokine production and inflammatory pathways.
Subjects who have elected to participate in the optional WAT biopsy will be identified previously (at the time of enrollment) and when they arrive to Labor and Delivery for their scheduled cesarean section. The fat biopsy will be obtained on all c-sections (based on staf avilability)".
Now to include subjects not delivering at MHMC recruited through advertisement and referrals. Pre delivery CRU visits will occur as stated above. For those outside MHMC facilities all attempts would be made to obtain the placenta weight. When subjects deliver at an outside hospital we will attempt to obtain the umbilical cord blood and placenta for scheduled c-sections. Newborn peapod measurements obtained within 7 days, with either vaginal or c-sections, when available.
We are removing the UUN-post OGTT and indirect calorimetry. Currently it is optional based on staff availabilty and the equipment being in working order. Rational this adds considerable time to the study visit and the equipment has been somewhat unreliable with the humidity issues in the room where the test is conducted. The results so far have yielded uneven results so the test is unreliable at this point and therefore is being removed.

Again, for those subjects who cannot tolerate the OGTT, then a fasting glucose test will be substituted without changing the outcome of the study.

8.7 Please attach study design/subject visit schedule here:

If you have an electronic schedule of study visits and/or procedures please attach here.

Name	Description
Table 6 | History	

View: 09-00 Inclusion/Exclusion Criteria

9.0 Inclusion/Exclusion Criteria:

9.1 What are the inclusion criteria? Put this information in bullet form:

Please list inclusion criteria.

*BMI (wt/ht2) > or = 25 at the first antenatal visit
*Gestational age at randomization between 8 weeks and 16 weeks based on clinical information and/or ultrasound prior to 20 weeks gestation
*No medical problems such as hyperlipidemia, hypertension, or pregestational diabetes
*Between ages of 18 and 40 years old.
*Non-smokers
*None of the subjects will be taking any medication which might affect glucose or lipid metabolism.
*Confirmed singleton pregnancy
*Women who either plan on having a cesarean section or vaginal delivery

9.2 What are exclusion criteria? Put this information in bullet form:

Please list exclusion criteria.

***Women with BMI (wt/ht2) < 25 at the first antenatal visit**
***If gestational age by ultrasound falls outside the 8 to 16 weeks gestation the subject will be excluded**
***Women with major medical problems such as hyperlipidemia, hypertension, or pregestational diabetes**
***Smokers, excluding marijuana use**
***Major fetal anomaly, mild renal abnormalities in the fetus will not be a reason for exclusion, known chromosomal abnormality will be an exclusion criteria, with the exception of balanced translocations or inversions that are not of clinical significance.**
***Regular intake of fish oil supplements (defined as greater than 500 mg per week within the last four weeks). This is due to the placebo group receiving fish oil outside of the study.**
***Daily use of nonsteroidal anti-inflammatory agents.**
***Allergy to fish or fish products, gluten intolerant (because the placebo contains wheat germ oil, which is not gluten free).**
***Women who are vegetarians and do not eat any fish.**
***History of spontaneous preterm delivery less than 36 wks or 2 kg in previous pregnancy. Medically indicated preterm births are not an exclusion.**
***Heparin use or known thrombophilia (thrombophilias include homozygous for Factor V Leiden).**
***Moderate or high titer IgG anticardiolipin antibodies or prolonged activated PTT or other indication of presence of lupus anticoagulant, homozygous for prothrombin gene (G20210A) mutation, antithrombin III deficiency.**
***Protein S (low levels outside of pregnancy) or Protein C deficiency.**
***Hyperhomocysteinemia (due to safety concerns because n-3 may affect bleeding time).**
***Hemophilias including von Willebrand's disease (because of safety concerns associated with the hemophilia treatment combined with the n-3 supplements).**
***Planned termination of pregnancy.**
***Current hypertension or current use of antihypertensive medication (including diuretics), due to increased risk of adverse pregnancy outcome.**
***Pregestational diabetes due to increased risks of affecting fetal growth. We will not exclude women who develop GDM during pregnancy but consider a sub-analyses of these women depending on the number of subjects. Known maternal medical complications: cancer (including melanoma but excluding other skin cancers).**
***Current hyperthyroidism if not adequately controlled.**
***Renal disease with altered renal function (serum creatinine > 1.5).**
***Epilepsy excluding pseudoseizures**
***Systemic lupus (not discoid lupus), scleroderma, polymyalgia rheumatica.**
***Active liver disease (acute hepatitis, chronic active hepatitis, persistently abnormal liver enzymes).**
***Platelet or red blood cell disorder (including idiopathic thrombocytopenia purpura, a history of alloimmune thrombocytopenia in a previous offspring, significant anemia due to hemoglobinopathy but not sickle cell trait. Iron deficiency anemia will NOT be an exclusion as long as the hemoglobin is > 8 gm/dl.).**
***Chronic pulmonary disease (asthma of any degree of severity is NOT an exclusion).**
***Structural, functional or ischemic heart disease. Neither mitral valve prolapse nor paroxysmal supraventricular tachycardia are considered exclusions.**
***Known HIV positive with viral load greater than 1,000 copies/ml or CD4 count less than 350/mm3.**
***Current or planned cerclage due to interference with the natural cause of delivery.**
***Illicit drug or alcohol abuse during current pregnancy excluding marijuana.**
***Participation in another intervention study that influences maternal and fetal morbidity and mortality, maternal nutritional intake or participation in this trial in a previous pregnancy.**
***At the time of birth, all infants will be evaluated by a pediatrician to make sure that they are healthy. Infants will be excluded from further study if they have any medical problems such as respiratory distress syndrome.**

***Infants will also be excluded if they have any problems that exclude them from having estimation of body composition, for e.g. birth weight less than 2 kg.**

9.3 How will subject eligibility be determined and by whom?
Eligibility will be determined at screening by the study personnel and then final determination of eligibility will be made upon consultation with potential subject by PI.
Eligibility will be based upon meeting the above criteria and then after initial "run-in" period, whether or not the subject has consumed at least 50% of the inert supplements that she has been given.

Please describe in detail.

9.4 Will you exclude women and minorities, or persons under 21 from enrollment?
No

Check yes or no.

9.5 If yes, which groups are you excluding? Provide justification for your decision.
Subjects under age 18 will be excluded only.
We will exclude women less than 18 years old from the study because of the detailed understanding of the study protocol required and the time commitment to complete the protocol.

List groups to be excluded then provide justification.

9.6 Attach Documents:

Name	Description
There are no items to display	

If you are unable to fit your answers in the text boxes provided please attach as a word document.

View: 10-00 Risk/Benefits

10.0 Assessment of Risk I:

10.1 Identify and distinguish between those procedures that are standard versus those that are experimental. Include the frequency and duration of each activity and the total length of subject participation:
All procedures are for research purposes, none are for standard care.

Please distinguish between those procedures that are standard versus those that are experimental. Describe in detail all experimental procedures.

Frequency and duration of each visit:

Time of recruitment:
Subjects will be recruited in the antenatal clinic/offices. All subjects eligible for the study will undergo a "run in period" of 1 week, if determined to meet eligibility, then subjects will be enrolled into the study.
This visit will take an estimated 15 minutes of time.

Study Visit Day 1:
Height and weight
Possible obstetrical ultrasound (if uncertain)
History and physical exam
Laboratory studies
2 hour 75g ISogtt or fasting glucose test
Food frequency questionnaire
Measure of body composition
Randomization to either placebo or n-3 PUFA supplementation
This visit will take an estimated 3 and a half hours of time.

Subsequent Prenatal Visits (every 2 to 4 weeks):
Subjects will return to the CRU after each scheduled clinic visit to bring back their pill packs to account for compliance, receive a portion of their stipend and new pill packs.
These visits will take an estimated 30 minutes of time.

Visit Day 2:
Subjects will return to the CRU between 34 to 36 weeks to repeat the protocol described in Visit Day 1, to determine the changes over time and between groups.
This visit will take an estimated 3 and a half hours of time.

Delivery:
For those pts delivering at an outside hospital, placenta collection will be obtained if possible. We would also attempt to get the umbilical cord blood on a scheduled c-section along with the placenta on any outside facility. All attempts will be made to schedule the infant for neonatal body composition within 7 days of birth.
This visit will take an estimated 30 minutes of time.
The WAT biopsy will be obtained at the time of cesarean delivery for the patients who elect or require cesarean delivery (and choose to participate in that Specfic Aim).

10.2 Describe any therapeutic alternatives to the research that may exist. How are they different from those procedures that subjects would normally undergo?
n/a

Describe any therapeutic alternatives. Can subjects receive this drug or device outside of a research study?

10.3 What are the outcome variables and how will they be analyzed? What are the statistical and analytical methods that will be used? *Note this section can be copied from the NIH Grant Application or from the Statistical and Analytical Methods section of the industry trial protocol.*
This revised grant application primarily focuses on the second specific aim of the original RO-1 application to assess the insulin sensitizing effects as well as the mechanisms of n-3 PUFA action on systemic and adipose tissue inflammatory responses. The new focus responds to the

Define outcomes and describe data analysis, please include a power calculation.

reviewer's primary concerns regarding the affect of n-3 PUFA supplementation to decrease inflammation during human pregnancy. We have modified our research protocol in order to recruit enough subjects to obtain meaningful data in a 2 year time. Based on our power analyses (see Table 1) by recruiting 120 overweight/obese women (60 placebo and 60 n-3 PUFA supplementation), assuming a 20% dropout rate, we will have sufficient power (50 subjects in each group) to assess the effect of n-3 PUFA to significantly reduce inflammation and insulin resistance in our pregnant population.
We will plan to recruit overweight/obese women in early gestation (<16 weeks gestation). Based on data from 2008 in our population over 330 women had an elective repeat cesarean delivery each year, greater than 70% of which were either overweight or obese. Hence not only will we be able to assess the effect of n-3 PUFA on systemic metabolic parameters but because elective cesarean delivery is considered a steady state condition, we will be also able to assess inflammatory gene expression in white adipose tissue (WAT) with minimal risk of labor related inflammatory changes.

See Table 1 attached below

Insulin sensitivity will be measured using the ISogtt method which based on our previous research with euglycemic clamps in pregnant women has the strongest correlation with insulin sensitivity. By combining the ISogtt with measures of free fatty aids and indirect calorimetry protocol, we will also assess the affect of n-3 PUFA on lipid metabolism and route of lipid oxidation. Of note, the shortened time period of the novel proposal (from 5 to 2 years) with a smaller number of study subjects will not allow to reach the statistical power to assess the effect of n-3 PUFA on fetal adiposity and placental inflammation. However, these data will be collected as noted in the original application (for e.g. Pea Pod estimation of neonatal adiposity) and placental tissue stored for later analysis.

In addition to maternal anthropometrics, metabolic parameters and plasma concentration of inflammatory cytokines this study will also characterize the inflammatory status of maternal white adipose tissue (WAT). This approach represents the mechanistic aspect of the revised proposal. Clinical use of n-3 PUFAs has recently emerged has a novel strategy to improve the metabolic consequences of obesity and inflammation through signaling adipose tissue molecules and transcription factors related to the PPAR family. We will analyze whether the anti-inflammatory of n-3 PUFA are related to decreased macrophage migration into the adipose tissue as well as their phenotype and subsequent activation in candidate signaling pathways.

see Study Design Diagram attached below

10.4 If the above requested information does not fit in the text box please attach a word document here:

If the requested information does not fit in the text box please attach a word document.

Name	Description
Study Design Diagram | History	
Table 1 | History	

View: 10-01 Risk/Benefits

10.1 Assessment of Risk II:

10.5 List and quantitate the risks involved for each experimental procedure in bullet form. Identify risks as common (greater than 10%) uncommon (greater than 1% up to and including 10 %) rare (1% or less). This must match the risks listed in the Consent Form:

Select all that apply.

***Height and weight: obtained as part of routine obstetrical care (risk = rare).**
***Obstetrical ultrasound: there are no risks associated with the used of ultrasound to measure dating of pregnancy (risk = rare).**
***History and physical exam: there are no risks associated with obtaining a medical history from the patient and performing a routine physical exam (risk = rare).**
***Laboratory studies: fasting blood will be obtained at the time the intravenous line is established for the IsOGTT in all possible cases (risk = rare).**
***2 hour 75g ISogtt: there are minimal risks in performing thsese studies in pregnant women. The OGTT has a minimal risk of thrombophlebitis, because of the use of an indwelling IV line to draw serial lab studies (risk = rare).**
***Indirect calorimetry: there are no risks associated with the indirect calorimetry (risk=rare).**
***Food frequency questionnaire: there are no risks associated with answering the questionnaire (risk = rare).**
***Measure of body composition (Bod Pod): there are minimal risks associated with the Bod Pod for pregnant women (risk = rare)**
***Pill counts: there are no risks to the subject from the pill counts (risk = rare).**
***Cord Blood: there are no risks to the subject from obtaining cord blood as the cord has already been removed from the neonate (risk = rare).**
***Placental Weight and Volume: there are no risks to the subject from measuring the weight and volume of the placenta, as they have already been removed from the subject (risk = rare).**
***Placental tissue biopsy: there are no risks to the subject from performing a placental tissue biopsy, as the placenta has already been removed from the subject (risk = rare).**
***Neonatal body composition (pea pod): there are minimal risks to the neonate in performing the pea pod (risk = rare).**
***Consumation of supplements: An n-3 PUFA supplement containing approximately**

850 mg of EPA and 882 mg of DHA has been used in previous studies after myocardial infarction. A total of 5,666 patients were assigned the supplement for 3.5 years. The drug showed a favorable safety profile, with the most frequently reported side effects being gastrointestinal disturbances (4.9%) and nausea. The safety concerns of exposing pregnant women to n-3 have been discussed by Olsen. According to Olsen, even though fish oil has been shown to increase bleeding time, the clinical relevance of this finding remains unclear. In his randomized controlled clinical trials involving a total of 2,150 pregnant women, Olsen found no significant difference in the bleeding times of the women receiving the fish oil supplement when compared to the controls. Despite this finding, Olsen has noted that in trials involving fish oil among pregnant women, women and infants, attention needs to be paid for any possible bleeding complications including vaginal bleeding and intracerebral hemorrhage. Reproductive toxicology studies of DHA-rich extracted oil were conducted in Sprague-Dawley rats and New Zealand White rabbits. Maternal exposure during organogenesis did not adversely affect the frequency of postimplantation loss, mean fetal body weight per litter, or external, visceral, or skeletal malformations in either the rat or the rabbit. In the rats, a no observed effect level (NOEL) was seen at all levels tested, including the highest level, 22 g/kg/day. In the rabbits, the NOEL for developmental toxicity was 1.8 g/kg/day. In another study of concentrates of DHA and arachidonic acid administered to pregnant rats during the period of organogenesis, there was no change in pre- or postimplantation losses, reasorptions, live births or sex ratios. There were no fetal malformations. The investigators concluded that these oils are not teratogenic at doses representing a 100-fold safety factor over expected use levels. (risk = rare).

10.6 **Are there defined stopping rules?** (•) **Yes** ( ) No

Describe in enough detail for the IRB to assess safety.

What are the stopping rules for the study? What are the conditions under which a subject will be withdrawn from the study for safety reasons, i.e. disease progression?
Subjects may withdraw from the study at any time, per the subject's request. We would stop the study if the subject were at risk from any unforseen medical complication, e.g., DVT (deep venous thrombosis) or obstetrical complication, i.e., abruptio placenta. These are normative obstetrical studies.

What findings, events, or conditions would require a research subject to be removed from the study? (i.e. disease progression)
None, we did the intent to treat approach.

10.7 **What Category of risk will study participants be exposed too?**

Should be consistent with risks listed in the Consent Form.

Name
Physical

10.8 **If Other listed above please specify:**
n/a

A text box is provided for further explanation.

10.9 **Describe the availability of medical or psychological services that participants might require as a consequence of participation in this the research:**
If the subject should so require, they will receive care from their primary obstetrical provider. Social services are available if needed as the patients are receiving obstetrical care at MetroHealth.

A text box is provided for further explanation.

10.10 **Describe in detail any measures in place to minimize or protect against the exposure of study subjects to these risks:**
*In order to minimize the minimal risk of local bruising, infection, or blockage of the vein during blood drawing, only trained, experienced personnel will perform these blood draws.
*For the possible risk during the oral glucose tolerance test (OGTT) of occasional nausea and very rare vomiting, the subject ingests the glucola in a reclining chair and can lay down if such a case occurs.

Discuss any provisions for intervention in the event of an Adverse Event i.e. stopping rules.

10.11 **Please add any documents related to the above questions:**

Name	Description
There are no items to display	

If your answers to the above questions are too long for the space provided please attach them here.

View: 10-02 Risk/Benefits

10.2 Benefits:

10.12 **Describe the potential benefits to the subject as a result of participating in this research. If there is no direct benefit to subjects please state that as well:** ***Note: payment or compensation to subjects for participation is not to be considered a potential benefit.***
The potential benefits to study subjects are that they will be made more aware of the physiological changes that occur in their bodies during pregnancy. Although these subjects will have assessment of maternal and fetal/neonatal metabolism, these studies may provide

Describe potential benefits to the study subjects.

the data necessary to plan for future specific diagnostic and treatment modalities during gestation in order to improve long term perinatal outcome.

10.13 Describe the potential benefits to society as result of this research:
The importance of the knowledge to be gained from the proposed research is the potential of obtaining a better understanding of the mechanisms involved in the development of increased fetal growth and adiposity which has occurred over the last 25 years in infants of obese women. The increase of obesity in all segments of the population is a grave public health concern. If we can better understand the mechanisms of increased fetal growth and adiposity then these studies may provide the data necessary to plan for future specific diagnostic and treatment modalities during gestation in order to improve both short and long term outcome.

Describe potential benefits to society.

10.14 What is the risk/benefit ratio of the research?
Because the risk to mothers and fetus/newborns from this research project are minimal, we expect the potential benefits of this study to outweigh the inconvenience of participating.

Discuss why the risks are reasonable in relation to the anticipated benefits.

10.15 Attach Documents:

Name	Description
There are no items to display	

Attach documents here.

View: 11-00 Study Participant Information I

11.0 Study Participant Information I:

11.1 How will the Principal Investigator assure he/she has access to a population that would allow recruitment of the required number of study participants (i.e. prep for research):
We will recruit overweight/obese subjects from women registering for obstetrical care prior to 16 weeks gestation. Subjects will be recruited through advertisement in the outpatient reception area, as well as from referral obstetrical care givers. The clinical research specialist will participate in recruitment of all study subjects and obtain written informed consent. The nature of the studies to be performed and the potential risks will be explained to each subject. All subjects are asked to read and sign a consent form approved by the Institutional Review Board at MHMC. Subjects will be informed that they may withdraw from the study at any time without endangering their medical care. Confidentiality will be maintained. There are approximately 3,500 deliveries performed each year at MHMC. Approximately 60 percent of our patients have pregravid BMI > 25. The primary cesarean section rate at MetroHealth is approximately 15%. The majority of these women are overweight/obese women. More than 60% of women with a prior cesarean section elect to undergo a repeat cesarean delivery rather than have a trial of labor. Based on these data, there should be little difficulty in recruiting 120 subjects over a 2 year period. These statistics also apply to the demographic characteristics of reproductive age women in Northeast Ohio.

How does the PI know he/she has the required number of subjects?

11.2

Please give the total #of subjects to be enrolled at all sites and anticipated subjects to be enrolled at MHS.

Anticipated number of subjects (all sites): [enter a number]
120

Anticipated number of subjects to be enrolled at MHS: [enter a number]
120 total, i.e., 60 randomized into each group.

Anticipated number of potential subjects to be approached: [enter a number]
120

11.3 If this is a multi-site study, how many sites will there be? [enter a number]
2 MHMC facilities

How many total sites?

11.4 Subject Characteristics:

Subject Population Categories
Normal Healthy Volunteers
Outpatients
Patients with the "disease in question"

Check all that apply

11.5 Subject Source:

Subject Source Characteristics
Subjects referred or recruited from other physicians practices
Public subject recruitment by advertisement, flyers, websites etc. (Note: required IRB review and approval)
Subjects identified from Medical Records or databases outside the Principal Investigator's department or group practice

Check all that apply

11.6 If "other" list above in either 11.4 or 11.5 please describe:

If applicable please describe.

View: 12-00 Study Participant Information II

12.0 Study Participant Information II:

12.1 Select age range of study participants: *Check all that apply.*

Subject Age Range
0 - 6
18 - 64

12.2 *Check yes or no.*

* Will the study enroll vulnerable subject groups?
Yes

* Will you be enrolling Children?
(•) **Yes** ( ) No

* Will you be enrolling Pregnant Women and/or Fetuses?
(•) **Yes** ( ) No

* Will you be enrolling decisionally impaired subjects?
( ) Yes (•) **No**

* Will you be enrolling Prisoners? ( ) Yes (•) **No**

12.3 Please identify any vulnerable populations participating in the study: *Check all that apply.*

Vulnerable Populations
Poor / Uninsured
Minors - Children under 18
Employees
Students
Pregnant Women
Non-English Speaking
Minorities

12.4 If you selected "other" above please describe: *Please describe other.*

12.5 If you are going to enroll <u>any</u> vulnerable populations please describe the safeguards you will put in place to protect these vulnerable Populations. *Please enter a detailed plan.*
Safeguards include full disclosure of the study and answering any questions related to the protocol. Assurances are made to the subjects that the decision as to whether or not to parrticipate in this study will not affect their treatment, employment, or education at MetroHealth.

View: Supplemental Review Form for Research with Children I

Supplemental Review Form for Research with Children I:

Federal regulations require the IRB to provide additional protections for children involved as subjects in research. There are four categories of permissible research that involve children [45 CFR 46.404, 46.405, 46.406, or 46.407]. **Please complete each section as it applies to your research. Each question must be fully answered, or your study will be returned and IRB review will be delayed.**

ASSESSING RISKS AND BENEFITS

When assessing risks and benefits, consider the variability in health status of the subjects to be enrolled, their medical experiences, and the extent to which the research procedures will be a burden to the subjects in the context of their daily lives and/or routine medical care. Be sensitive to how a procedure that generally entails little to no physical or psychosocial risks may affect someone with limited (or no) understanding of the situation. Procedures that usually present no more than minimal risk to a healthy child include: physical exam, ultrasound, urinalysis, obtaining a small amount of blood, EEGs, allergy scratch tests, minor changes in diet or daily routine, and/or the use of standard psychological or educational tests. The assessment of the probability and magnitude of the risk, however, may be different in sick children and may vary depending on the diseases or conditions the subjects may have.

Minimal Risk

: As defined in the regulations 45 CFR 46.102(i), "*minimal risk*" means the probability and magnitude of harm or discomfort anticipated in the research are not greater in and of themselves than those ordinarily encountered in daily life or during the performance of routine physical or psychological examinations or tests."

Answer 1, 2, 3, or 4 below if applicable:

1. **Is this Pediatric Research not involving greater than minimal risk (see definition above) [45 CFR 46.404]:**
   ( ) Yes (•) **No**

Explain Why you think this study is not greater than minimal risk:
Omega 3 Supplemental studies are performed on pregnant women all across the country and they have even put DHA in prenatal vitamins because other studies have found that it's beneficial for the baby's brain development. Umbilical cord blood and obtaining placenta tissue poses no risk to the baby because this is obtained after the baby is born. There are no known risks to measuring the baby's body composition with the Pea Pod device. Measuring the baby's height, weight, and fat thickness will not hurt the baby.

****

2. **Is this Pediatric Research involving greater than minimal risk but presenting the prospect of direct benefit to individual subjects [45 CFR 46.405]. Answer all questions below.** ○ Yes ◉ **No**

Explain why you think the procedures or interventions are of greater than minimal risk:

Describe the anticipated or possible health benefits, and explain why you think the study interventions or procedures hold out the prospect of direct benefit to each individual subject:

Explain why you think the risk to subjects is justified by the anticipated benefit:

Explain why you think the risk/benefit ratio is at least as favorable to the subjects as that presented by available alternative approaches:

****

3. **Research involving greater than minimal risk and no prospect of direct benefit to individual subjects, but likely to yield generalizable knowledge about the subject's disorder or condition. [45 CFR 46.406]. Answer all questions below:**
○ Yes ◉ **No**

Explain why you think the interventions or procedures represent no more than a minor increase over minimal risk:

Explain why you think the interventions or procedures present experiences to subjects that are reasonably commensurate with those inherent in their actual or expected medical, dental, psychological, social, or educational situations:

Explain why you think the interventions or procedures are likely to yield generalizable knowledge about the subjects' disorder or condition that is of vital importance for the understanding or amelioration of the subjects' disorder or condition:

****

4. **Is this Pediatric research that is, not otherwise approvable that presents an opportunity to understand, prevent, or alleviate a serious problem affecting the health or welfare of children [45 CFR 46.407]. This category includes research that does not meet the criteria for any of the above three risk/benefit categories. Research in this category represents more than a minor increase over minimal risk and no prospect of direct benefit to subjects.**
○ Yes ◉ **No**

Explain why you think the research presents a reasonable opportunity to further the understanding, prevention, or alleviation of a serious problem affecting the health or welfare of children:

NOTE: This kind of research requires review by the Secretary of DHHS, after consultation witha panel of experts in pertinent disciplines, and public review and comment. Contact the Manager and Chair of the MHS IRB at 216-778-2077, if you believe your research falls into this category.

View: Supplemental Review Form for Research with Children II

Supplemental Review Form for Research with Children II:

SECTION 2: PERMISSION OF PARENTS/GUARDIANS AND ASSENT OF CHILDREN

5.
Permission of Parents or Guardian (check one below)

INSTRUCTIONS:

*Check **all** that apply and answer the related questions.*

☐ The permission of both parents or a guardian(s) will be sought unless one parent is deceased, unknown, incompetent, or not reasonably available, or when only one parent has legal responsibility for the care and custody of the child (required for categories 45CFR.406 & 407 see questions [3] and [4]).

or

☑ The permission of only one parent will be sought (acceptable for categories 45CFR46. 404 & 405 see questions [1] and [2]).

6.
Assent of Children (check one below)

☐ The assent of each child who is capable of providing assent based on age, maturity, and psychological state will be sought.

or

☑ The assent of each child will not be sought because the capability of all of the children in this study population is so limited that they cannot reasonably be consulted.

Explain why the capacity is so limited, e.g., age, maturity and/or psychological state:
The assent of each child is not sought because of their age (newborn) and we obtain permission from the parent's and no further testing is done on them beyond newborn state.

or

☐ The assent of each child will not be sought because the intervention or procedure involved in the research holds out a prospect of direct benefit that is important to the health or well-being of the children and is available only in the context of the research.

Explain what the direct benefit may be and why it is only available in the context of the research:

NOTE: *Ohio*

law generally permits individuals to consent for their own medical care at age 18, but limited exceptions permit individuals to consent at an earlier age to certain treatments. Consult the MHS Policy I-34 on the MIV.

View: Supplemental Form for Research on Pregnant Women and Fetuses I

Supplemental Form for Research on Pregnant Women and Fetuses I:

Federal regulations require the IRB to provide additional protections for pregnant women and human fetuses involved in research [45 CFR 46.204]. **Please complete each section as it applies to your research. Each question must be fully answered or the form will be returned, and IRB review will be delayed.**

ASSESSING RISKS AND BENEFITS

When assessing risks and benefits, consider the variability in health status of the subjects to be enrolled, their medical experiences, and the extent to which the research procedures will be a burden to the subjects in the context of their daily lives and/or routine medical care. Procedures that usually present no more than minimal risk include: urinalysis, obtaining a small amount of blood, EEGs, allergy scratch tests, minor changes in diet or daily routine, and/or the use of standard psychological or educational tests. The assessment of the probability and magnitude of the risk, however, may vary depending on the diseases or conditions the subjects may have.

Minimal Risk

: As defined in the regulations 45 CFR 46.102(i), "*minimal risk*" means the probability and magnitude of harm or discomfort anticipated in the research are not greater in and of themselves than those ordinarily encountered in daily life or during the performance of routine physical or psychological examinations or tests."

SECTION 1: RESEARCH INVOLVING PREGNANT WOMEN OR FETUSES

Pregnant women or fetuses may be involved in research if

all of the following conditions listed below are met [45 CFR 46.204]. **Please provide study-specific information with your explanations.**

1.) Where scientifically appropriate, pre-clinical studies, including studies on pregnant animals, and clinical studies (including studies on non-pregnant women) have been conducted and provide data for assessing potential risks to pregnant women and fetuses.
Yes

2.) Provide a brief description of relevant prior pre-clinical and clinical studies, and based on this information, what you think the risks are for pregnant women?
Fish oil supplementation has been used extensively in non-pregnant and pregnant women for various medical benefits. Most recently, the Maternal-Fetal Medicine Network used a fish oil supplementation which we will be using in this protocol to assess the affect of fish oil on the risk of prematurity. Although the fish oil supplementation was not found to be efficacious in the prevention of preterm delivery, there were no side effects noted. Hence, at this time we feel that fish oil supplementation would be an appropriate avenue of research based on our specific aims.

Based on prior pre-clinical and clinical studies, there are minimal risks to pregnant women regarding fish oil supplementation. We have elected to include extensive exclusionary criteria listed in this application, which would help exclude patients who may have potential issues with fish oil supplementation.

3.) Provide a brief description of relevant prior pre-clinical and clinical studies, and based on this information, what you think the risks are to the fetuses?
To the best of our knowledge, there has been no evidence of any side effects in the fetus regarding fish oil supplementation in pregnancy.

4.) The risk to the fetus is caused solely by interventions or procedures that hold out the prospect of direct benefit for the woman or the fetus; or, if there is no such prospect of direct benefit, the risk to the fetus is not greater than minimal (see definition above) and the purpose of the research is the development of important biomedical knowledge which cannot be obtained by any other means. (•) **True** ( ) False

5.) Explain what direct benefit may accrue to the women taking part in this research, or to the fetuses:

The direct benefit that may accrue to the women taking part of this research is the decrease in insulin resistance in obese pregnant women mediated by a decrease in inflammation. The benefit to the fetus is the potential decrease of adipose tissues which has both short and long term implications.

OR, if there is no prospect of direct benefit to the women, or to the fetus

6.) Explain why you believe the risk to the fetus is not greater than minimal risk:

7.) Explain what new important knowledge will be gained, and why the information could not be obtained by any other means:
It is important to conduct this study in the human as the human fetus is unique in the amount of adipose tissue that is accrued during pregnancy. With the increase in obesity in the population, there has been an increase in fetal growth, particularly fetal adiposity, which has both short and long term risk. Non-human primates would not be a good model since at birth, the amount of body fat in a non-human primate is approximately 2-3% whereas in the human fetus, it is anywhere between 12-14%.

8.) Any risk is the least possible for achieving the objectives of the research:
(•) **True** ( ) False

9.) Explain how the risks have been minimized to the least possible to achieve the research objectives:
We have used standard dose fish oil supplementation that has been used in other clinical studies that have been approved by the NIH and there have been no adverse effects. Additionally, based on information provided in the Background section, there is a significant decrease in PUFA n-3 Omega's in pregnant women because of the FDA's recommendation to limit fish consumption in pregnancy because of the potential risk of mercury toxicity. Hence, fish oil supplementation is already being used by many women and this study will control for this, check the levels and have a data safety monitoring committee. For all these reasons we believe that this study has the potential to provide benefits for both mother and fetus at minimal risk.

View: Supplemental Form for Research on Pregnant Women and Fetuses II

Supplemental Form for Research on Pregnant Women Fetuses II:

SECTION 2: INFORMED CONSENT

(Check all that apply)

9.) Informed consent will be obtained in writing from the pregnant woman in accordance with the consent provisions of federal regulation 45 CFR 46.116.
◉ **True** ○ False

10.) When the research holds out the prospect of direct benefit solely to the fetus, informed consent will be obtained in writing from the father in accordance with the consent provisions of 45 CFR46.116, except, if he is unable to consent because of unavailability, incompetence, or temporary incapacity, or the pregnancy resulted from rape or incest. (see MHS IRB Guidelines on establishing Paternal Availability on the IRB Home Page) ◉ **True** ○ False

11.) Each individual providing consent will be fully informed regarding the reasonably foreseeable impact of the research on the fetus. This information is included in the MHS Consent Form Guidelines. ◉ **True** ○ False

SECTION 3: City of Cleveland Law states:

Section 231.05 Experimentation upon a Fetus
No person shall experiment upon or sell the product of human conception which is aborted, irrespective of the duration of the pregnancy.
(Ord. No. 1861-A-73. Passed 12-10-73, eff. 12-10-73)

Section 609.02 Abortion Offenses
(a) Abortion Defined. As used in this section "abortion" means the purposeful termination of a human pregnancy by any person, including the pregnant woman herself, with an intention other than to produce a live birth or to remove a dead fetus or embryo. Abortion is the practice of medicine or surgery for the purposes of RC 4731.41. (RC 2919.11)

(b) Abortion Without Informed Consent Prohibited.

(1) No person shall perform or induce an abortion without the informed consent of the pregnant woman.

(2) No person shall knowingly perform or induce an abortion upon a woman who is pregnant, unmarried, under eighteen years of age and unemancipated except as authorized under RC 2919.12.

(3) Whoever violates this section is guilty of unlawful abortion, a misdemeanor of the first degree unless the offender has previously been convicted of or pleaded guilty to a violation of division (b) of this section or RC 2919.12. (RC 2919.12)

(c) Abortion Trafficking.

(1) No person shall experiment upon or sell the product of human conception which is aborted. Experiment does not include autopsies pursuant to RC 313.13 and 2108.50.

(2) Whoever violates this section is guilty of abortion trafficking, a misdemeanor of the first degree.
(RC 2919.14; Ord. No. 1414-86. Passed 11-3-86, eff. 11-5-86)

SECTION 4: CONFIRMATION OF PRINCIPAL INVESTIGATOR

12.) No inducements, monetary or otherwise will be offered to terminate a pregnancy:
◉ **True** ○ False

13.) The individuals engaged in the research will have no part in any decision as to the timing, or procedures used to terminate a pregnancy: ◉ **True** ○ False

14.) The individuals engaged in the research will have no part in determining the viability of the Neonate: ◉ **True** ○ False

15.) This research will be conducted in accordance with the provisions of all local, state and federal laws: ◉ **True** ○ False

View: 13-00 Recruitment I

13.0 Recruitment I:

All external advertisements (for radio, print media or TV) must be approved by MHS Communications Department prior to submission to the IRB so the IRB can see the final advertisement or script. All Advertisements on the MIV or On Hold messaging must be approved by the IRB before they are placed. You may not advertise a study which is not approved by the IRB. Please note that all studies which have a contract which an external sponsor must have that contract signed before any advertising can be done.

13.1
Recruitment Methods/Sources: *Check all that apply.*

Name
Advertisements-Newspapers/Magazines, Television, Radio
Notices/Posters/Flyers
Letters

13.2 If "Other" checked in 13.1 please explain: *Please explain what other means.*

13.3 Describe in detail all recruitment strategies for each subject group (as listed in Section 11.0) selected for this research: *Please describe recruitment strategies in detail.*

We will recruit overweight/obese subjects from women registering for obstetrical care prior to 16 weeks gestation . Subjects will be recruited through advertisement in the outpatient reception area at MHMC, as well as from outside referral obstetrical care proveders. We will do this by sending out a provider referral letter along with a brochure of our study to outside obstetrical care givers of which, in turn, patients may contact us if they would like more informatin about the study and are interested in participating. Along with this, we will have other IRB-approved advertisements from which to recruit @ MHMC facilities, ie., poster board from which we can set up tables with our brochure, and approved articles discussing the benefits of Omega-3. Also, recruit with an IRB-approved 'On Hold Messaging' while people who contact MHMC can listen to while they are on hold.

13.4 What measures will be taken during the recruitment process to safeguard against the potential coercion or appearance of coercion of human subjects, particularly vulnerable subject groups? *Please give an explanation of safeguards to be used.*

It will be stressed upon the subjects that participation in the study is strictly voluntary and that they may withdraw their consent at any time. It should also be noted that the PI has been performing studies with pregnant women for over twenty years, and the project coordinator has been doing them for over eleven. We feel extremely confident that not only the way that we recruit these patients, but the manner in which we carry out these studies is in no way coercive to the subjects. Furthermore, we do not offer large stipends that may be construed as coercive in and of themselves.

The letter and brochure will only be sent out to subjects who have shown an interest in participating and have been initially approached or referred by their provider.

13.5 Incentives to Subjects: Will subjects receive any incentives (payments, free service, gifts, etc.) for participation in the research? *This information must mirror the consent form language.*

Yes

13.6 If yes, please describe these incentives and how they will be disbursed: *Note: payment or compensation to subjects for participation is not to be considered a potential benefit.* *Describe incentives, if they are to be pro-rated based on visits completed please give that information. This information must mirror consent form language.*

The subjects will be compensated $100 for participation. They will receive, in the form of a check, $40 after the first and after the second set of studies in the CRU. The subject will then receive the additional $20 after the baby is measured at delivery.

13.7 Please attach copies of all recruitment/advertising materials and verbal scripts: *Attach copies of all recruitment and advertising materials.*

Name	Version
Are You Pregnant Poster | History	0.03

Name	Version
Article: Omega-3 Fish Oil During Pregnancy | History	0.01
Article: Prenatal Omega-3 May Aid Babies Brain Development | History	0.01
Instruction Sheet for the One Week Run-In | History	0.02
Instruction Sheet for the Randomization of Study Drug | History	0.02
Local Paper Advertisement | History	0.01
No-show Correspondence | History	0.02
Omega 3 Brochure PDF copy | History	0.09
Omega 3 Letter | History	0.02
Omega On Hold Message Radio Ad | History	0.01
Provider Referral Letter | History	0.02
Research Study Announcement. 5th version | History	0.06
Scheduling Correspondence | History	0.02
Spanish Omega Brochure | History	0.02
Track Change Copy of Brochure.June 2010 | History	0.06
Track change of Research Study Announcement | History	0.05
Track Change to Are You Pregnant Poster | History	0.01
Track Changes of Visit Reminder Letter | History	0.01
Visit Reminder Letter | History	0.04

View: 13-01 Recruitment II

13.1 Recruitment II:

13.8 Expense to Subjects: Will subjects incur any expenses as a result of participation in the study or will they be billed for any study-related procedures?
No

Check yes or no, make sure this information is in the consent.

13.9 If yes, please describe the expenses or charges that subjects will be assessed:

Please provide information regarding expenses to subjects and add information to consent.

13.10 Compensation For Injury: If applicable, will funding be available to compensate subjects for injuries sustained as a result of participation in this research?
No

Check yes or no, make sure this information is in the consent.

13.11 Who will cover the costs related to any injuries sustained due to participation in the study?
The subject or the subject's health insurance will be responsible for covering these costs.

Please describe in detail. Examples subjects or their insurance company, study sponsor.

View: 14-00 Data Collection

14.0 Data Collection:

14.1

A. What type of data will you be collecting as part of this research?

Existing data must be in place or on the shelf prior to the submission of the research protocol to the IRB.

Prospective data is collected in real time.

Will you collect existing data?

or

Will you collect prospective data?
○ Yes ○ No

or

Will you collect both existing and prospective data? ● **Yes** ○ No

***Definitions:* Data are considered to be existing data only if they were in place or "on the shelf" prior to the submission of the research protocol to the IRB. Data are considered prospective if they are created and collected as part of the research i.e. from surveys, questionnaires.**

Tell the IRB why you are collecting this data i.e. to verify inclusion criteria.

B. Why are you collecting this data?

What will be the purpose of collecting and/or reviewing the data (new data or existing data).
The purpose is to validate the data relating to the index pregnancy, for example the gestational age at the time of the termination, any medications that the subject may be taking at the time of the study, and any medical problems that the subject has.

14.2

If you are collecting existing data:

Specify the types of existing data you will use in this study.

Specify the type(s) of existing data sources you will use (medical records, school records, publicly available records, existing database). If you are collecting data from an exisitng database and that database contains PHI, you must provide the IRB Approval letter (attach to Section 27.00 Additional Documents).
We will be using medical records and EPIC chart for purposes of obtaining information pertaining to the current pregnancy, i.e, dating of the pregnancy, as well as a medical history.

Time frame i.e. last 10 years or from 1990-2000.

What is the timeframe of the existing data you wish to review? (i.e. 2000-2006)
Any information from the start of the index pregnancy, which is the pregnancy for which the subject is being enrolled into the study. For example, an ultrasound that has been obtained to confirm the dates of the pregnancy.

14.3 **If you are collecting prospective data:**

Where or how will the data be obtained? (i.e. surveys, questionnaires, psychological tests)
The information will be collected from the medical record and questionnaires. For all MHMC patients the information will be obtained from EPIC (when available) and questionnaires. For all non-MHMC study patients after a release of records is signed a copy of the medical record will be obtained from her provider.

Where will data be obtained? i.e. survey.

14.4 **How will the data you collect be identified?**

Types of Data Identification:

Please select how your subject data will be identified.

Name
Deidentified/Confidential- Data will be linked to subject(s) via a code or indirect identifier (i.e. study IDs or numbers)

14.5 **Will the information collected from these records be linked to any research subjects by identifiers? (i.e. name, MRN#, DOB)**
(•) **Yes** ( ) No

Will your data be linked to subjects?

Please answer questions about the security of the data in section 15.00

14.6 **If subject data will be deindentified using a code will there be a link or a key? Please describe. Who will have the key and where will the key be kept?**
Data will be deidentified in the following manner. A code will be kept by the KeyMaster Todd Sarbach in Redcap. There will be no copies of the code maintained anywhere else. The study staff will have access to the key in Redcap as they are actively enrolling subjects. Once the study is completed and the database is locked for data analysis. Only the Key Master Todd Sarbach will be able to reidentify the Name, MRN# and DOB. The IRB will be asked for permission to access the code if it becomes necessary to re-identify a subject or subjects.

Explain how Data will be linked.

Under the HIPAA Regulations Keys must be stored separately from your data. Keys may no longer be kept as paper files they must be electronic. The MetroHealth KeyMaster is Charles Sarbach Please contact Mr. Sarbach at 216-778-3330 or csarbach@metrohealth.org. He will assist Investigators in developing a Key in the MetroHealth RedCap database. He can also assist investigators to develop a database for their study. There is no charge for either of these services it is provided as part of the CASE CTSA.

14.7 **Data Collection Form(s):**

Name	Version
There are no items to display	

Add data collection forms and CRFs.

View: 15-00 Data Security I

15.0 Data Security I:

It is imperative that the IRB is proactive and consistent in protecting all research data containing Protected Health Information(PHI).

15.1 * Are the records for this study (some or all) electronic? (•) **Yes** ( ) **No**

What is Protected Health Information? The Privacy Rule protects certain information that covered entities use and disclose. This information is called protected health information (PHI), which is generally individually identifiable health information that is transmitted by, or maintained in, electronic media or any other form or medium. This information must relate to 1) the past, present, or future physical or mental health, or condition of an individual; 2) provision of health care to an individual; or 3) payment for the provision of health care to an individual. If the information identifies or provides a reasonable basis to believe it can be used to identify an individual, it is considered individually identifiable health information.

The following questions must be answered when submitting a new protocol.

15.2 * Are you collecting PHI? (•) **Yes** ( ) No

15.3 Is any PHI going to be stored as paper files? (●) **Yes** ( ) No

15.4 Is any PHI going to be stored in an electronic file format? (i.e. access, excel) (●) **Yes** ( ) **No**

15.5 Is your data being stored on a laptop computer? ( ) Yes (●) **No**

15.6 Will you be using RedCap to store your data? (●) **Yes** ( ) **No**

Which RedCap Database will you be using?

Name
MetroHealth

15.7 Are you planning to store your data using a portable storage device?(i.e. jump drive, external hard drive, cd) ( ) Yes (●) **No**

**Per current MetroHealth Policy PHI may not be stored on portable electronic devices.*

15.8 Are there any circumstances under which you would want to remove data from MHS? (i.e. take data home to work on it) Give details below. Please note identified data can't be removed from MHS unless there is permission granted in the HIPAA Authorization. If you are unsure about what is identified data please consult the IRB staff. If you feel you will need access to your data when you are off campus you should ask the MHS IT Department located in Rammelkamp room R 134 about VPN access.
( ) Yes (●) **No**

If you answered yes to question 15.8, please explain?

15.9 Where will the records pertaining to this research be stored? (give the actual physical location of the paper records i.e building name and room number); and/or the secure network drive where the data is being stored.
Paper records will be kept in the research office G272, 2nd floor Bell Greve, 2500 Metrohealth Drive, Cleveland, OH 44109. Electronic records will be secured on the MHS network 'G' drive and 'S' drive and Redcap.

State the exact physical location of paper files and the network drive for electronic files.

15.10 How will these records be secured (we are refering to both paper records and electronic records)? Examples for electronic records (i.e. secure drive, password protected documents, encrypted jump drive). Examples for paper records, must be double locked (i.e. locked office and locked file cabinet or a locked file box inside a locked cabinet).
Paper records are kept in a locked file cabinet in a locked office. Electronic records are kept or stored on a secure MHS network drive and Redcap.

i.e. locked cabinet, locked room.

15.11 Who will have access to the data?
N/A

Give name and title exclude study staff who are MHS employees.

Please Note: All study documents must be retained for a minimum of four years after study completion (even when no subjects have been enrolled), twenty-two years if study involves children or pregnant women. Records for device studies must not be assigned a destruction date until the FDA approval status is determined, at which point records will be retained according to the scheme above (minimum of four or twenty-two years as appropriate). Under HIPAA regulations you must keep a record of all medical records where you looked at or recorded PHI (without a HIPAA Authorization) for 6 years (i.e. prep for research).

MHS Record Retention Policy VII-4

15.12 How long will you keep the records pertaining to this research? Where will these records be stored after the study has been completed?
Records will be kept for 22 years after completion of the study. Paper records are kept in a locked file cabinet in a locked office, G272. Electronic records are kept or stored on a secure MHS network drive and Redcap.

Check the MHS Record retention policy for guidance.

You must have a plan for data destruction.

15.13 Where, when, and how will the information be destroyed?
After 22 years the data will be destoyed per MHS policy/protocol.

*Please Note: There are EPA regulations surrounding the destruction of CDs, DVDs, Floppy discs and other portable storage media. If you want to destroy these types of media please contact Ron Wallace in Environmental Services at 778-4776.

View: 15-01 Data Security II

15.1 Data Security II:

15.14 Who (non-study staff) will have access to the records? Give name and title of individuals. Where an individual's name is not known give title i.e. monitor from CRO.

List all those not study staff who will see and have access to data.

15.15 Will data be transmitted to the sponsor? ○ Yes ◉ **No**

Are you sending CRFs to sponsor?

15.16 If yes, describe what data will be sent to the sponsor and the provisions that have been made for preservation of confidentiality in the transmission of data to the sponsor:
n/a

Please describe i.e. will you be using encryption software?

15.17 Will the data from this research project be transmitted to anyone other than the sponsor? ○ Yes ◉ **No**

Check yes or no.

15.18 If yes, to whom will this data be transmitted?
n/a

Please describe organization or individual.

15.19 Describe the data that will be sent to entities other than the sponsor and what provisions have been made for the preservation of confidentiality:
n/a

Please describe data, and confidentiality provisions.

View: 16-00 Request for a Partial Waiver of HIPAA Authorization

16.00 Request For a Partial Wavier of HIPAA Authorization

An IRB, under certain circumstances, may allow researchers to forgo obtaining an authorization; this is called a waiver of authorization. A waiver of authorization may be full or partial:

- full waiver: an IRB waives the requirement for authorization for all uses of PHI for a particular research protocol (see Section 16.01 Request for a Waiver of HIPAA Authorization);
- partial waiver: an IRB waives the requirement for an authorization only for some uses of PHI for a particular research protocol. Researchers are required to obtain subjects' Research Authorizations after recruiting and enrolling subjects via a partial waiver and prior to creating or using PHI during research procedures.

Partial Waiver for Preparatory for Research Activities:
According to HHS guidance on the Privacy Rule the preparatory to research provision permits covered entities to use or disclose protected health information for purposes preparatory to research, such as to aid study recruitment. **However, the provision at 45 CFR 164.512(i)(1)(ii) does not permit the researcher to remove protected health information from the covered entity's site.** *As such, a researcher who is an employee or a member of the covered entity's workforce could use protected health information to contact prospective research subjects.* The preparatory research provision would allow such a researcher to identify prospective research participants for purposes of seeking their Authorization to use or disclose protected health information for a research study.

Under the preparatory to research provision, a covered entity may permit a researcher who works for that covered entity to use PHI for purposes preparatory to research. A covered entity may also permit, as a disclosure of PHI, a researcher who is not a workforce member of that covered entity to review PHI (within that covered entity) for purposes preparatory to research.

16.1
Are you requesting a Partial Waiver of HIPAA Authorization? ◉ **Yes** ○ No

Why are you requesting a Partial Waviver?

Check yes or no.

16.2
Is the purpose of the Partial Waiver Recruitment (including screening of Medical Records)?
◉ **Yes** ○ No

Is the purpose of the Partial Waiver to request access to PHI for Non-MetroHealth personnel?
○ Yes ◉ **No**

Check yes or no.

16.3 Will the use of Protected Health Information (PHI) involve more than minimal risk to the privacy of the patients?
○ Yes ◉ **No**

Check yes or no.

16.4
The IRB as part of it's review of this request must have certain reassurances that Patient Privacy will be protected, please respond to the following questions true or false.

Check true or false.

1.) The PHI will be used solely to facilitate the research protocol as an aid to study recruitment or to expand the research study. The waiver would allow identification of prospective research participants for the purpose of seeking authorization to use or disclose PHI for a research study. Essentially, PHI will be used to identify and contact potential research participants. Only contact and screening information (race, age, medications, diagnosis, and primary physician) will be recorded, and no information will leave the premises of MetroHealth Medical Center. The information will not be disclosed outside the research group for this study. (•) **True** ( ) False

2.) Information about potential subjects who are not interested in participating will be destroyed after the patient declines enrollment. The information of patients choosing to participate will be further used to schedule an appointment. As soon as the research staff sees the participant, a full authorization will be obtained to collect, use and disclose PHI for the remainder of the study. (•) **True** ( ) False

3.) The PHI will not be reused or disclosed. Because the PHI belongs to individuals who are not yet in the study, oversight provisions do not apply. After subjects are formally enrolled, an authorization will be in effect and the waiver will no longer apply. (•) **True** ( ) False

16.5 If you did not answer true to all three parts of question 16.4 please explain:

Please explain your response to any statement where you have entered false.

16.6 Please give a detailed explanation as to why this research activity cannot be practicably conducted without a Partial Waiver or without access to PHI:
Our study population has an extensive listing of inclusive and exclusive criteria that has to be met. And, we rely on the EMR to screen for potential candidates prior to study staff approaching them in clinic.

Example: our study population has xxx disease and we rely on the EMR information to identify and contact potential subjects.

16.7 Who will have access to PHI? Please list below:

Name	Employer	Department	Employer Name
Patrick Catalano	MHS	Obstetrics/Gynecology	The MetroHealth System
Sharon Groh-Wargo, PhD	MHS	Pediatrics	The MetroHealth System
Sylvie Hauguel-de Mouzon	MetroHealth	OBGYN	The MetroHealth System
Gregory Kitagawa	MetroHealth Medical Center	OB/GYN	The MetroHealth System
Stephen Myers	Metrohealth	OB/GYN	The MetroHealth System
Larraine Presley	MHS	Ob/Gyn	Obstetrics/Gynecology
Shoi Smith	MetroHealth System	OB/GYN Research	Obstetrics/Gynecology

Add the names of persons who will have access to PHI.

16.8 Are you or anyone who assists you Non-MetroHealth Personnel? ( ) Yes (•) **No**

Check yes or no.

**Note all Non-MetroHealth Personnel have to go through employee orientation, have a security clearance and Epic training before they can access the MetroHealth EMR. Also all all Non-MetroHealth Personnel must work under the control of a member of the MetroHealth Staff.*

If you have previously completed an MHS **Prep for Research form** add that form here:

If you filed a Prep for research form with IT and RABO please attach it here.

Name	Version
There are no items to display	

Partial Wavier Memos completed prior to 11/26/2010 will populate here.

Old Memos Requesting Partial Waivers (prior to 11/26/2010):

There are no items to display

View: 16-01 Request for a HIPAA Waiver of Authorization

16.1 Request For a HIPAA Waiver of Authorization:

16.9 **Are you requesting a Waiver of HIPAA Authorization?**
No

Check yes or no.

Check one, if you check no then hit continue and go to the next page.

If you are requesting a Waiver In order for the IRB to Grant a Waiver you must answer questions 16.10-16.16

16.10 Disclosure of Protected Health Information (PHI) will not involve more than minimal risk to the privacy of the patients/subjects:

Check true or false.

16.11 What is the plan to protect patient/subject identifiers from improper use and disclosure?

i.e. This unique identifier will be used on the data collection form. Only the PI will have access to the key linking the unique identifier to patient/subject names.

16.12 What is the plan to destroy the identifiers at the earliest opportunity consistent with the conduct of the research?

i.e. The unique identifier key will be retained in Red Cap and will be destroyed two years after the study ends.

16.13 Will PHI be reused or disclosed to others:

Check yes or no.

16.14 Please complete the following: Data will only be used to analyze...

i.e. Data will only be used to analyze...

16.15 Describe why this research can not be conducted without a waiver:

i.e because many of the subjects who participate in this treatment are dead or have transferred to other treatment modalities, or are transient. To obtain HIPAA Authorization from these individuals would be a greater risk to their loss of privacy.

16.16 Describe why this research could not be conducted without access to and use of PHI:

i.e. It would not be possible to determine linkages between ....and clinical outcomes without the use of PHI.

View: 16-02 HIPAA II

16.2 HIPAA II:

16.11

Which of the following identifiers about subjects will be collected for this study?

Check all that apply, your answers will help the IRB to determine if your data is a limited data set.

Name
2. Telephone Numbers
3. Address - Street
4. Address - Town or City
5. Address - State
6. Address - Zip Code
8. Names or Initials
16. Medical record or prescription numbers
20. Social Security Numbers
21. Dates (except year) related to an individual (birth date, admission date, discharge date, date of death)

*These Questions deal with the collection of data and data use agreements. If you are **not** receiving data or sending data out to another entity this does not apply to you. If you have a signed contract with a sponsor or are in a cooperative group that has a signed agreement with MHS this does not apply to you. Data use agreements specify the conditions under which data can be shared between MHS and other organizations or individuals.*

16.12 If you have selected only numbers 4, 5, 6, or 22 in question number 16.11 your research is considered to use a limited data set. If either of the following conditions apply, you will need to obtain a Data Use Agreement and complete a waiver of authorization or obtain a HIPAA authorization from the subjects. (check one):

Name
There are no items to display

Check one, please read carefully if you are not receiving data or sending data out to another entity this does not apply to you, move on to 16.14. If you have a contract with a sponsor or you are in a cooperative group that has a signed agreement with MHS this does not apply to you. In all other cases please contact Kim

Bauchens at 778-8526 or Bonni Kurtz at 778-5219 with questions about data use agreements.

16.13 Attach a copy of the Data Use Agreement:
Name Description
There are no items to display

Attach Data Use Agreement.

View: 16-03 HIPAA III

16.3 HIPAA III:

16.14 If any other unique identifying number, characteristic or code is selected, please specify:
n/a

Please specify this question refers back to the list of 22 identifiers.

16.15 If a link to an identifier will be used (i.e. code numbers) is selected, please describe the coding mechanism that will be used:
n/a

Describe the coding mechanism.

16.16 Will a certificate of Confidentiality be obtained for this study? No

Check yes or no.

16.17 If yes, please attach a copy the Certificate of Confidentiality:
Name Version
There are no items to display

Attach a copy of the Certificate of Confidentiality.

16.18 Describe how you will protect the privacy of participants. Describe specifically how you will gather information from or about them. ***Please note while confidentiality concerns data, privacy concerns people. Example People may be uncomfortable answering questions about their employer in an open cubicle, so investigators may arrange for a more private location.***
The confidentiality of research material obtained from study subjects will be maintained throughout the study period. Each subject's file will be kept in the P.I.'s office. As well, data collection will be captured onto Metrohealth Redcap (Research Electronic Data Capture), a web-based database application on secured servers within the Metrohealth data center. The investigators will have access to the PHI for 22 years, at which time it will be destroyed (this is written on the informed consent). Reference to a particular subject in any public communication will be either by use of the subject's initials or study number. All pertinent research information will be available to the study subject and their health care provider upon request of the subject. Review of relevant data, for example plasma glucose concentrations, will be made available at the time that the particular research study is completed.

Please note while confidentiality concerns data, privacy concerns people.

View: 17-00 Waiver of Informed Consent

17.0 Request for a Waiver or Alteration of Informed Consent:

17.1
Are you requesting a Waiver of Consent [45 CFR 46.116(d)] OR a Waiver of Documentation of Consent [45 CFR 46.117 (c)].
Yes

Answer yes or no.

If no hit continue button and you will go to the next page.

If yes please Note:

Note: Waivers of consent are not applicable if the research is subject to FDA regulations, except the following.

FDA Exception from general requirements:

1. Emergency Ues: Waivers of Informed Consent in FDA-regulated studies are permissible in case of life-threatening situations, inability to communicate, not sufficient time and no alternative method, even if research presents more than minimal risk [21CFR50.23];
2. Planned Emergency Research: If the study satisfies the requirements under 21CFR50.24 "Exception from Informed Consent Requirements for Emergency Research."

17.2
Waiver of Consent: If you are requesting a waiver of consent, please provide the justification and address each of the following points for the IRB's consideration:

Check true or false.

This research study involves no more than minimal risk:

Note: practicably does not mean it would be inconvenient.

The waiver will not adversely affect the rights and welfare of the subjects:

This research could not practicably be carried out without a waiver:

Whenever appropriate, the subjects will be provided with additional pertinent information after participation:
Yes No

17.3 **Please explain your answers to the above questions (You must provide the IRB with enough information to make a decision):** *Please explain in detail.*

An IRB may **waive the requirement to obtain a *signed* consent form** for some or all subjects if it finds either of the conditions below. In cases in which the documentation requirement is waived, the IRB may require the investigator to provide subjects with a written statement regarding the research.

17.4 **(1) The only record linking the subject and the research would be the consent document and the principal risk would be potential harm resulting from a breach of confidentiality. Each subject will be asked whether the subject wants documentation linking the subject with the research, and the subject's wishes will govern; OR** *Check true or false.*

Check yes or no.

17.5 **(2) The research presents no more than minimal risk of harm to subjects and involves no procedures for which written consent is normally required outside of the research context.**
Yes No

17.6 **If you are requesting any Alteration to the standard consent form/process (written long form consent is the standard) please provide a detailed explanation or plan.** *Example of an alteration: verbal consent.*

We are including a consent document to obtain the father of the baby's verbal consent when he is not otherwise available for consenting. We will obtain phone permission from the father of the baby with 2 research staff and make any attempts for him to sign the informed consent as he is able to. Uploaded track copy and clean consent copy to section 17.16

View: 17-01 Informed Consent Process I

17.1 Informed Consent Process I:

17.7 **Who will be approached to obtain consent/assent:** *Check all that apply.*
Consent Method
Subjects will be asked to sign a study consent form after receiving a complete explanation of the study.

Idendtify all Staff obtaining consent on page 1 question 1.4 by selecting the corresponding role.

17.8 **Subject Comprehension: What measures will be taken to ensure that subjects fully understand the nature of their involvement in the research?** *Please give brief explaination.*

Note to Investigator:

To address issues of comprehension on the part of the participant or representative, and who is involved in obtaining consent, the answers to following questions should be addressed:

1.) Once a potential participant is identified, what process is followed to inform the subject of the study prior to obtaining a signature on the informed consent form?

a. Who introduces the study to the potential subject?

b. Who reviews the informed consent document in depth?

c. Do you require the potential participant to have another person present during the presentation of the study?

2.) Who answers the questions presented by the potential participant and/or family?

3.) What method is used to determine if the potential participant fully understands the study, what is required from them, risk and benefits, and their rights as a participant?

4.) Is the principal investigator usually present during the presentation of the informed consent?

1) The clinical research specialist will explain the study in detail to the potential subject, along with potential risks involved in the study.
a. The study will be introduced to the potential subject by the potential subject. If the subject is referred by a practitioner, it may be very briefly introduced by the practitioner, but will be discussed in detail, by the clinical research specialist.

b. The approved consent form will be reviewed by the Clinical Research Specialist. Subjects will be asked to read the consent form. All questions will be asked and then consent will be obtained.
c. Another person will not be required to be present during the presentation of the study to the potential participant.
2) Questions presented by the potential participant and/or family will be addressed by the Clinical Research Specialist. Any questions that cannot be addressed by the Clinical Research Specialist will then be answered by the PI.
3)If there is doubt as to the potential participant's comprehension of the study, what is required of them, the risks and benefits, or their rights as a particpant, we will ask the potential participant to repeat them to us to determine comprehension. If we determine that the subject does not comprehend any of the above, the potential subject will not be enrolled into the study.
4)The PI will not typically be present during the presentation of the informed consent, but meet with the subject at the end of the run-in period of the protocol (randomization).

17.9 Capacity to Consent: How will capacity to consent be assessed? *This question is to be addressed for all subjects not just those with limited decision making capacity. Identify who will make this assessment? Suggested language....all subjects will be awake, alert and oriented, be able to read etc. It is important to address issues like ability to read and understand information in the consent.*

How will you determine capacity to consent?

Subjects with limited capacity, such as minors, will not be enrolled in the study. If a subject cannot read, she will also not be enrolled. Assesment may be made by the individual obtaining consent with the final determination to be made by the PI as to the appropriateness of the enrollment of the subject.

17.10 Attach a description of the Consent Process: Explain the process of obtaining consent from subjects. Under what settings and conditions will consent be obtained? What will be the timing/waiting period? What measures will be taken to ensure that subjects will make decisions independently? *Note to Investigator: The "informed consent process" should include sufficient time for the participant to review and consider participating with the assistance of family members, research partners or representative if necessary. Other items to consider regarding time / waiting periods are: Is the potential participant given a copy of the consent form to read prior to the discussion of the study? Is it presented in person or mailed (where they can review it in the privacy of their own home)? How much time elapses between the presentation of the study and informed consent form and the actual signing of the form? The answers to these questions will ensure the PI has considered this component of the process and will reassure the IRB that the PI is allowing adequate time for the participant to make an informed decision and minimize the possibility of coercion or undue influence.*

Attach a plan for consenting subjects. This must give detail about the consent process.

Name	Description
Consent Changes- Track Changes June 2011 | History	
Consent Process-Clean Copy June 2011 | History	

17.11 Parental Permission and Youth Assent: Complete this question only if enrolling minors. How will parental permission and youth assent (if applicable) be obtained?
not applicable

Give details of assent process and assent form.

View: 17-02 Informed Consent Process II

17.2 Informed Consent Process II:

17.12
What method will be used to document the consent process (i.e. a note in EPIC)? Not how you will get consent only how you will document consent has been obtained, i.e chart note, note in study file.
The original signed consent form will be filed in the patient's chart in Medical Records, a copy will be present to the CRU and a copy will be kept in the subject's research chart in the Research Office. A signed copy will also be given to the subject. A note will be charted in the subject's EPIC chart that written informed consent was obtained by the PI.

i.e chart note, note in study file.

17.13 What type of Informed Consent will be used in this study? (check all that apply):

Consent Type
Verbal Consent
Written/Signed Consent by Subject

Check all that apply

A non-return cover memo applies to a study in which you are sending out a questionnaire with a memo or letter that informs participants about the study but does not need to be signed and returned. If they complete and return the questionnaire they have given consent.

17.14 **If other, please specify:**

If other, please give specifics.

****Attach all consent forms (Informed Consent, Genetic Consent and HIPAA) here:****

17.15 **Please attach a copy of each Informed Consent form(s) and HIPAA Authorization you are using for this study:**

Attach Consent form(s) and HIPAA Authorization here

Name	Version
November 2010 Track Changes Copy	0.13
November 2010Consent Clean Copy	0.19
Omega Father of the Baby Verbal Consent Clean Copy April 2011	0.01
Omega Father of the Baby Verbal Consent Track Copy April 2011	0.01
Omega-3 HIPPA November 2009	0.04
Spanish Omega Consent	0.02
Spanish Omega HIPAA	0.02
Withdrawal from study letter	0.01

17.16 **Will non-English speaking subjects be enrolled?**

Check one

(•) Yes ( ) No Clear

If the answer to 17.17 is no we will not be enrolling non-English speaking subjects then tell the IRB why not?

Please give the IRB an explanation as to why non -English speaking subjects will not be enrolled.

17.17 **If non-English speaking subjects will be enrolled please provide information about the person(s) obtaining consent (what language they will speak)and how you will deal with written translation(s):**

Provide information about translating consents and having interpertative services available for consent.

We will intend to translate the consent form into Spanish. We will try to recruit a Clinical Research Specialist who speaks both English and Spanish.

View: 18-00 Data Safety Monitoring I

Section 18.0 Data Safety Monitoring Plan

DATA AND SAFETY MONITORING PLAN GUIDE

WHEN DO YOU HAVE TO COMPLETE A DATA SAFETY MONITORING PLAN?

FOR THE IRB- **All interventional studies that are greater than minimal risk should have a Data Safety Monitoring Plan. The IRB reserves the right to require a Data Safety Monitoring Plan for any study.**

Archived IRB Data Plans - prior to 9/28/2010

Although this is a minimal risk study, we will have a DSMB to review the study on a semi-annual basis. They consist of the following:
Dr. John Moore - Pediatrician
Dr. Dianne Schubeck - Obsetrician
Dr. Edith Lerner - Nutritionist at CWRU

FOR THE CRU- ALL CRU PROTOCOLS [**Recent NIH guidelines stipulate that all protocols that involve human subjects, a signed consent form and are conducted on, or use the resources of, the CTSA Clinical Research Unit - MHMC (CRU) are required to have a Data and Safety Monitoring Plan (DSM Plan).]**

What is a Data and Safety Monitoring Plan (DSM Plan)?

A DSM Plan is a prospectively defined strategy to assess the assumptions made in the trial design while the study is in progress. Its main purpose is to ensure the safety of participants in clinical research studies and the validity and integrity of research data collection. A properly designed DSM Plan improves the scientific quality and yield from a clinical trial and the protection of human subjects.

The DSM Plan needs to address the nature of the safety monitoring and who will be conducting that monitoring. It may be reasonable for a single individual to perform the monitoring in a small trial with minimal/low risk while a local independent or an external data and safety monitoring board (DSMB) may be required for more complex/high risk trials.

Key elements to be incorporated in a DSM Plan

- Assessment of risks and monitoring level
- Safety contact: Who is responsible?
- Safety monitoring: Who will do it? How often?
- Informed consent process; consistency with the protocol
- Data collection process
- Adverse Events Monitoring: Anticipated and unanticipated
    - Description of anticipated adverse events
    - Grading and attribution method
    - Reporting of unanticipated adverse events
    - Plans for periodic reporting
    - Impact on termination of subjects from the study and study closure

Step 1 - only for Investigators Using the CRU:

1.A Is your protocol approved and supported by the Ireland Cancer Center? ○ Yes ◉ No

If Yes - The Comprehensive Cancer Center Data and Safety Moniotoring Plan for Clinical Trials is on file. Proceed to Step 5.1.B If No, Proceed and complete Steps 2-5

****

Step 2 - all Investigators - Provide Information in order to determine the level of safety monitoring required

2.A List all data collection types and study procedures (this information will pull from Section 8 Methods and Procedures questions 8.1, 8.2, 8.3)

Data Collection types:

Name
Banking of Specimens/Data
Chart Review - Prospective
Existing/Retrospective Data/Specimens
Interviews, questionnaires or psychological tests
Anthropomorphic evaluations

Social-Behavioral Procedures:

Name
Surveys/Questionnaires

Medical Procedures:

Name
Study of Human Biological Materials (i.e. Urine Collection)
Study of Existing Data
Medical Tests, Comparisons, Evaluations
Clinical Assessments (EEG, EKG, SCID, etc.)
Venipuncture (Blood Draw)
Anthropomorphic Measurments

****

****You must select the risk level Please read the information below, check the applicable boxes and select an appropriate risk level.***

Level I: Minimal and Low Risk Studies (examples of studies that are minimal and low risk studies)

Types of Studies:

Name
Chart Review, interview, questionnaire
Anthropomorphic evaluations

Level II: Moderate Risk Studies (examples of studies with populations, drugs, and procedures that are moderate risk)

Types of Studies:

Name
Pregnant Population

Level III: High Risk Studies (examples of diagnostic procedures and drugs or device studies which are high risk)

Types of Studies:

Name
There are no items to display

2.B If you do not see your study procedures on the above list please add in the procedures being done for research purposes:

Add additional risk(s):

Procedure	DSMB Risk
There are no items to display	

Select the Appropriate Level of Risk for this study based on the criteria above:

Level of Risk:
Risk Level I Mimimal and Low Risk Studies

Now Select the appropriate Level of Monitoring and give your justification:

2.C Rank Level of Monitoring (select one by checking the box)

Minimal/Low/Moderate Levels of Monitoring ☑

Justification for selecting Minimal/Low/Moderate Level of Monitoring Required:
As defined in the regulations 45 CFR 46.102 (i) "minimal risk" means the probability and magnitude of harm or discomfort anticipated in the research are not greater in and of themselves than those ordinarily encountered in daily life or during the performance of routine physical or psychological examinations or tests." Pregnant women undergo OGTTs during normal screening in their pregnancy.

High Level of Monitoring ☐

Justification for selecting Risk High Level of Monitoring:

View: 18-01 Data Safety Monitoring II

18.01 Data Safety Monitoring II

A designee will perform the safety monitoring:
◉ **Yes** ○ No

Identify the designee [provide contact information]:
The study coordinator: Larraine Presley, ext 8-8927; pager # 3151.

A medical monitor or independent individual/safety officer will be performing the safety assessments.

(•) **Yes** ( ) No

Identify who will be performing the safety assessments [provide contact information]:
The DSMB as listed below meet with the Omega team semi-annually and more frequently if a serious adverse event occurs. The Principle Investigator reviews the data and the study coordinator initiates the report. The DSMB (who is independent of the study) files the DSMB report.

Has a Data Safety Monitoring Board or Committee been established for this study?

(•) **Yes** ( ) No

Identify these members by name, title and qualifications. How often will the DSMB meet? How frequently will the DSMB report it's findings?) data prior to 9/28/2010 read only.
Although this is a minimal risk study, we will have a DSMB to review the study on a semi-annual basis. They consist of the following:
Dr. John Moore - Pediatrician
Dr. Dianne Schubeck - Obsetrician
Dr. Edith Lerner - Nutritionist at CWRU

We are in the process of confirming the above board and once in place we will notify the IRB.

If there is a DSMB or DSMC is it a nationally constituted Data and Safety Monitoring Committee? ( ) Yes (•) **No**

Please enter the Name of Contact or Chair, Address and Phone or E-Mail:

Is there a locally constituted Data and Safety Monitoring Committee or Board that will perform the safety monitoring. Specify composition and responsibilities in the box below. *Note: Board Members should not have conflicts with this study or with study personnel.*

( ) Yes (•) **No**

Names of Members of Local DSMB [provide contact information]:

3.B.1 Description of anticipated adverse events. Pulled from question 10.5.

*Height and weight: obtained as part of routine obstetrical care (risk = rare).
*Obstetrical ultrasound: there are no risks associated with the used of ultrasound to measure dating of pregnancy (risk = rare).
*History and physical exam: there are no risks associated with obtaining a medical history from the patient and performing a routine physical exam (risk = rare).
*Laboratory studies: fasting blood will be obtained at the time the intravenous line is established for the IsOGTT in all possible cases (risk = rare).
*2 hour 75g ISogtt: there are minimal risks in performing thsese studies in pregnant women. The OGTT has a minimal risk of thrombophlebitis, because of the use of an indwelling IV line to draw serial lab studies (risk = rare).
*Indirect calorimetry: there are no risks associated with the indirect calorimetry (risk=rare).
*Food frequency questionnaire: there are no risks associated with answering the questionnaire (risk = rare).
*Measure of body composition (Bod Pod): there are minimal risks associated with the Bod Pod for pregnant women (risk = rare)
*Pill counts: there are no risks to the subject from the pill counts (risk = rare).
*Cord Blood: there are no risks to the subject from obtaining cord blood as the cord has already been removed from the neonate (risk = rare).
*Placental Weight and Volume: there are no risks to the subject from measuring the weight and volume of the placenta, as they have already been removed from the subject (risk = rare).
*Placental tissue biopsy: there are no risks to the subject from performing a placental tissue biopsy, as the placenta has already been removed from the subject (risk = rare).
*Neonatal body composition (pea pod): there are minimal risks to the neonate in performing the pea pod (risk = rare).
*Consumation of supplements: An n-3 PUFA supplement containing approximately 850 mg of EPA and 882 mg of DHA has been used in previous studies after myocardial infarction. A total of 5,666 patients were assigned the supplement for 3.5 years. The drug showed a favorable safety profile, with the most frequently reported side effects being gastrointestinal disturbances (4.9%) and nausea.

The safety concerns of exposing pregnant women to n-3 have been discussed by Olsen. According to Olsen, even though fish oil has been shown to increase bleeding time, the clinical relevance of this finding remains unclear. In his randomized controlled clinical trials involving a total of 2,150 pregnant women, Olsen found no significant difference in the bleeding times of the women receiving the fish oil supplement when compared to the controls. Despite this finding, Olsen has noted that in trials involving fish oil among pregnant women, women and infants, attention needs to be paid for any possible bleeding complications including vaginal bleeding and intracerebral hemorrhage. Reproductive toxicology studies of DHA-rich extracted oil were conducted in Sprague-Dawley rats and New Zealand White rabbits. Maternal exposure during organogenesis did not adversely affect the frequency of postimplantation loss, mean fetal body weight per litter, or external, visceral, or skeletal malformations in either the rat or the rabbit. In the rats, a no observed effect level (NOEL) was seen at all levels tested, including the highest level, 22 g/kg/day. In the rabbits, the NOEL for developmental toxicity was 1.8 g/kg/day. In another study of concentrates of DHA and arachidonic acid administered to pregnant rats during the period of organogenesis, there was no change in pre- or postimplantation losses, reasorptions, live births or sex ratios. There were no fetal malformations. The investigators concluded that these oils are not teratogenic at doses representing a 100-fold safety factor over expected use levels. (risk = rare).

Additional Comments on anticipated adverse events:

3.B.2 Safety data/procedure used to preform evaluation:

Data to be evaluated:

Name
Subject interview and/or contact
Subject's vital signs
Subject's physical exam
Subject's symptoms or performance status
Clinical Test(s) (e.g. labs, ECG)

Who will evaluate safety data:
The DSMB as listed above meet with the Omega team semi-annually and more frequently if a serious adverse event occurs. The Principle Investigator reviews the data and the study coordinator initiates the report. The DSMB (who is independent of the study) files the DSMB report.

Frequency of Monitoring:

Name
6 Months

3.C. Grading method and attribution for adverse event reporting:

Grading method and attribution for adverse event reporting

The PI must identify what scale will be used to grade adverse events (AEs) and indicate his/her attribution/assessment of the relationship between the adverse event and the protocol/intervention. Each protocol may have a unique approach to grading adverse events and the PI should consult the parent protocol and/or funding source for specific grading scales. Suggested guidelines for the grading of adverse events are available below:

Example A: *Cancer Therapy Evaluation Program (CTEP) Common Toxicity Criteria (CTC II) available for viewing at http://ctep.info.nih.gov (see "Reporting Guidelines, Common Toxicity Criteria")*

Example B: Common grading scale

0 No adverse event or within normal limits or not clinical significant
1 Mild AE, did not require treatment
2 Moderate AE, resolved with treatment
3 Severe AE, resulted in inability to carry on normal activities and required professional medical attention
4 Life threatening or disabling AE
5 Fatal AE

3.C.1 Identify the scale to be used to Grade AEs in this study:

CRU Safety Scale:

Name
AEs will be graded using another system (specify and attach description).

3.C.2 Identify the attribution scale to be used in this study:

CRU Attribution Scale:

Name
The PI will determine the relationships of AEs to test procedure/device/agent as not related, possibly related, or definitely related, using standard criteria for clinical trials.

3.D. Population being studied: (populated from your answers to Sections 11.00 and 12.00)

Vulnerable subject groups? Yes

Children? Yes

Decisionally Impaired Subjects? No

Pregnant Women and/or Fetuses? Yes

Will you be enrolling Prisoners? No

Other Populations being studied:

Vulnerable Populations
Poor / Uninsured
Minors - Children under 18
Employees
Students
Pregnant Women
Non-English Speaking
Minorities

*** Note More Frequent monitoring intervals may be needed for vulnerable populations.***

4.A. Plan for Adverse Event Reporting:

All Reportable Events (Anticipated and Unanticipated events) from this protocol must be submitted using the MHA eIRB Reportable event form in a timely maner consistent with MHS IRB SOPs.

In addition to the MHS IRB adverse events and Uanticipated problems will be reported to:

Reporting Institutions (check all that apply):

Name
National Institutes of Health (NIH)
Food and Drug Administration (FDA)

If other has been selected above please specify:

4.B Stopping Rules or Conditions under which Subjects can be removed from the Study [this information is from Section 10.01 of the Protocol Risks/Benefits Questions

Are there defined Stopping Rules? Yes

What are the stopping rules for the study? Subjects may withdraw from the study at any time, per the subject's request. We would stop the study if the subject were at risk from any unforseen medical complication, e.g., DVT (deep venous thrombosis) or obstetrical complication, i.e., abruptio placenta. These are normative obstetrical studies.

What findings, events, or conditions would require a research subject to be removed from the study? (i.e. disease progression)
None, we did the intent to treat approach.

4.D. Additional Information (if Applicable):

Provide any other information relevant to the data and safety monitoring plan that was not already incorporated into this form.

Attach A copy of your Data Safety Monitoring Plan or other relevant information related to this form:

Name	Version
There are no items to display	

View: 19-00 Use of Human Biological Materials In Research I

19.0 Use of Human Biological Materials In Research I:

19.1 *Check yes or no.*

Will Human Biological Materials be collected as part of this study? (i.e. blood, tissue, fluids and substances etc.)
Yes

If no, hit continue and you will be taken to the next page.

**19.2**	**Will the storage or transportation of study materials place anyone at a health risk? In other words, are these biohazardous materials? Will they put the staff collecting them or transporting them at risk?**<br>**No**	*Check yes or no.*
**19.3**	**If yes, please explain:**	*Please explain the risks. Above and beyond universal precautions.*
**19.4**	**Will information from the materials be stored in an electronic database?**<br>Yes	*Check yes or no.*
**19.5**	**If yes, list the database(s) where the information from the materials will be stored and who will have access to them:**<br>This information will be stored in the CRU database which will be coded with subject identifiers. CRU staff and study staffonly will have access to the data. The data will also be stored in a database in the Research Office. Only the Investigators and the study staff will have access to this database.	*List the database(s) and who will have access to them.*
**19.6**	**Human Biological Material Destruction: please describe the plan for materials destruction (when, where, how and by whom):**<br>We anticipate that there will be no discard material, all samples will eventually be used in analyses.	*Give the destruction plan i.e. shipped back to sponsor for destruction at end of study, incinerated by Browning Ferris 3 months after study ends.*
**19.7**	**Storage of Human Biological Materials: please describe where, how and for how long the materials will be stored:**<br>All materials are stored in a -20 or a -80 degree departmental freezer until used for analysis. They will be stored there as long as we continue to do the analyses on these samples or until the material is completely utilized. We estimate that they will be used most likely within 10 years.	*Physical storage of materials where will it be, how will it be stored and for how long.*

View: 19-01 Use of Human Biological Materials in Research II

19.1 Use of Human Biological Materials In Research II:

**19.8**	**Does this research involve human cell/lines and/or products that are made from human biological materials?** (●) **Yes** ( ) No	*Check Yes or No.*
	**If yes, please explain:**<br>Serum plasma used in both CRU#1 and CRU #2 and umbilical cord blood will be stored in the -20 freezer. Along with this the placenta tissue used for analysis of tissue macrophages, assessment of the effects of n-3 PUFA supplementation on the secretory phenotype of resident macrophages.	*Please explain.*
**19.9**	**Will Human Biological Materials (tissue, blood or salavia) be collected in this study for genetic research?**<br>No<br><br>**If Yes, please provide additional discussion of the genetic testing components including who will conduct the tests:**	*Check Yes or No.*
**19.10**	**If yes, can subject(s) decide not to participate in the genetic research and still participate in the study?**<br><br>**Please submit the appropriate genetic consent/tissue storage form and attached at 17.15**<br>A template for this form can be found on the IRB Home Page. Note: if tissue storage is mandatory for participation in a study the subject consent must be included in the body of the consent form; if it is not mandatory it can be included as a separate page at the end of the consent form.	*Check Yes or No.*
**19.11**	**Will NIH Genome-Wide Association Studies (GWAS) be conducted?**<br>( ) Yes (●) **No**	*Check Yes or No.*
**19.12**	**Will you be sending samples/data to the NIH GWAS?** ( ) **Yes** (●) **No**	*Check Yes or No.*
**19.13**	**Will you be using sample/data obtained frm the NIH GWAS?** ( ) Yes (●) **No**	*Check Yes or No.*
**19.14**	**Please provide justification for using NIH GWAS:**	*Please explain.*

If this is a GWAS study you will need to submit a **Patient Information Sheet (add at 17.16).** This sheet should summarize the Genetic research component of this study and tell the subjects where their biological materials will be sent, what analysis they will undergo, who will have control of them and for how long and who to contact if they want to withdraw their permission. It must be clear to subjects that these samples will not be housed at MHS nor will the MHS Investigator retain control over them.

View: 20-00 Drug Information I

20.0 Drug Information I:

20.1

* **Does this study involve drugs?** Yes

If you check no and hit continue you will go to the next page.

If you are doing a drug study you may be required by law to register that study at Clinical Trials.gov Section 113 of the FDA Modernization Act mandates registration with ClinicalTrials.gov of investigational new drug efficacy trials for serious diseases or conditions. For more information click on the link below:
http://prsinfo.clinicaltrials.gov/registering.pdf

If you answer no and hit continue you will go to the next section.

Does this study involve:
An approved drug for a non-approved purpose

Is the study drug(s) FDA approved for this indication? ○ Yes ◉ **No**

Does this study involve use of a Placebo? ◉ **Yes** ○ No

Does this study have a drug washout period? ○ Yes ◉ **No**

Do you have an IND? ○ Yes ◉ **No**

If yes please give the IND: (include a copy of the FDA approval letter at 20.4)

Who is the sponsor or holder of the IND?

Does this study have an IND exemption? (include a copy of the FDA exemption letter at 20.4) ○ Yes ◉ **No**

20.2 **Fill in an entry for all drugs that will be used in the study:**

Please give a complete list.

Drug Name	FDA Approved (yes, no)	IND Number	Supplied By
Omega-3	yes		Eminent Services Corporation

20.3 **Manufacturer (name, address):**
Eminent Services Corporation
7495 New Technology Way
Frederick, MD 21703

Answer only if produced commercially.

20.4 **Attach a copy of:**
1.) Investigator Brochure and/or Package Insert
2. FDA Form 1571 Investigational new Drug Application Form
3.) FDA Form 1572 Statement of the Investigator Form
4.) FDA Correspondence (i.e. FDA Approval Letter for IND, FDA Exemption letter)

Attach the IB, 1572 and 1571 here.

Name	Description
Eminent Corporation | History	
package insert | History	

View: 20-01 Drug Information II

20.01 Drug Information:

20.5

Provide Drug Preparation and Administration information:
The study drug treatment goup involves Omega-3: containing 800mg DHA and 1200mg of EPA. Participants that are enrolled in the study will be randomized on their Visit 1 to the CRU, at which time they will be randomly placed on either the reserach study medication or the control group placebo. Whether they are place into either group, they will take the capsules as follows: They are to take 4 capsules on a daily basis> 2 capsules in the morning with food; and 2 capsules in the evening with dinner. They will continue to take the capsules up until the time that they deliver.

Please provide detailed information on how you will dose study subjects along with a dosing schedule and Pharmacy Manual.

Provide a Dosing schedule and a Pharmacy Manual:

Name	Description
There are no items to display	

Please answer yes or no.

20.6 Does this research study involve the dispensing of drugs on an outpatient basis?

(●) **Yes** ( ) No

IF you have answered No please hit continue and go to the next page.

20.7 Is the intent of the investigator to dispense and coordinate the drugs involved in this study?

(●) **Yes** ( ) **No**

If you do not plan to use the Research Pharmacy answer yes to this question.

20.8 Where the drugs will be stored and who will have access to them?

The dietary supplement will be supplied and shipped by Eminent Services Corporation out of Frederick, MD directly to Dr. Catalano's office so that it is not 'hung up' on some shipping dock, exposed to extreme temperatures.It will be kept in a locked cabinet of the study coordinator's office. This system has been checked out through our inpt pharmacy of which the lock system and chain of custody has been checked out and authorized by pharmacy via the form, appendix A. A daily temperature log with a Min/Max thermometer will be maintained to ensure that the temperature does not reach above 77 degrees. Only the study personnel and the Investigators will have access to them.

Please tell the IRB where the drugs will be stored and who will have access to them.

20.9

IF Yes, The primary investigator or designated study staff must notify the MetroHealth Pharmacy Investigational Drug Service (MPIDS) by providing them with a copy of the investigator's brochure(s) and the study protocol. The MPIDS pharmacist will ensure the investigator can comply with the required storage and distribution plan and return a signed copy of the "Investigator Responsibility for Research Medication Form" The signed form must be provided to the IRB at the time of study submission.

Please review the Pharmacy Policies on the MIV Section Q on Investigational Drugs.

Link to form:

Print this form out and have it completed and signed by PI.

Attach Appendix A signed by Investigator: Link to Form Attachment A

Name	Description
Appendix A.pdf | History	

View: 21-00 Medical Device Information I rev

21.0 Medical Device Information I:

Definition of a Medical Device:

An instrument, implement, machine, contrivance, implant, in vitro reagent, or other similar or related article, including a component part, or accessory which is

- Intended for use in the diagnosis of disease or other conditions, or in the cure, mitigation, treatment or prevention of disease in man or other animals.
- Intended to affect the structure or any function of the body of man or other animals, and which does not achieve any of its primary intended purposes through chemical action within or on the body of man or other animals and which is not dependent upon being metabolized for the achievement of any of its primary intended purposes.

In short any health care product that does not achieve its primary intended purposes by chemical action or by being metabolized. Medical devices include, among other things, surgical lasers, wheelchairs, sutures, pacemakers, vascular grafts, intraocular lenses, and orthopedic pins. Medical devices also include diagnostic aids such as reagents and test kits for *in vitro* diagnosis (IVD) of disease and other medical conditions such as pregnancy.

21.1

Is this a Medical Device Study? No

Answer yes or no.

If you are doing a device study you may be required by law to register that study at Clinical Trials.gov Section 113 of the FDA Modernization Act mandates registration with ClinicalTrials.gov of investigational new device efficacy trials for serious diseases or conditions. For more information click on the link below:

http://prsinfo.clinicaltrials.gov/registering.pdf

If you answer no and hit continue you will go to the next section.

21.2 Medical Device Generic Name: *Give generic name.*

21.3 Medical Device Brand Name: *Give brand name.*

Medicare Code Number:

21.4

As stated in regulations 21 CFR 812.3(m), a device may be considered a, Significant Risk Device, if it meets any of the following criteria and a determination is made by the IRB that the device presents a potential for serious risk to the health, safety or welfare of a subject.

21.5 Is this device intended as an implant? *Check one*

21.6 Is device to be used in supporting or sustaining human life? *Check one*

21.7 Is the device for use of substantial importance in diagnosing, curing, mitigating, or treating disease or otherwise preventing impairment of human health? *Check one*

21.8 Does this device present a potential for serious risk to the health, safety, or welfare of a subject? *Check one*

21.9 If you answered NO to all the above, or if an initial risk assessment has determined that this is a non-significant risk device (21CFR 812.3), attach the appropriate documentation for this justification: *Attach justification.*

Name	Description
There are no items to display	

21.10 What is the regulatory status of the study device? *Provide information on the regulatory status of the device.*

21.11 What is the long term plan for device management once the study closes? *Provide information such as how you will communicate important information to subject; plan for maintenance/repairs; contact information besides the PI.*

View: 22-00 Clinical Trials Registration

22.0 Clinical Trials Registration:

Note: Phase 2 - 4 trials of drugs and biologics (controlled clinical investigations other than Phase 1 investigations of a product subject to FDA regulation) AND trials of devices (controlled trials with health outcomes, other than small feasibility studies and pediatric post-marketing surveillance) must be registered per the Food and Drug Administration Act of 2007; NIH encourages registration of all trials, regardless of whether required under applicable law.

How are study protocols submitted to ClinicalTrials.gov?
The FDA Guidance Document (March 2002) (http://www.fda.gov/cder/guidance/4856fnl.htm) describes the submission criteria. The NLM has developed the Protocol Registration System (PRS), a Web-based tool for submitting information to ClinicalTrials.gov. Study sponsors or their representatives may register online to apply for a PRS account (http://prsinfo.clinicaltrials.gov/).

22.1 Has this trial been registered on www.clinicaltrials.gov? *Web link to clinical trails website.*
(•) **Yes** ( ) No

22.2 If **Yes**, who registered the trial?(i.e. sponsor, investigator) *Please respond*
Investigator

22.3 Please provide ClinicalTrials.gov Identifier (i.e. NCT00391872) *The sponsor can provide you with this information or you can look it up on the website.*

NCT00957476

22.4 If **No**, are there plans to register the study? (•) **Yes** ( ) No *If you answer No you must provide an reason why this study will not be registered.*

22.5 If the answer to 22.4 is **No**, provide and explanation: *Provide a response if the answer to 22.4 is No.*

View: 23-00 Interview/Focus Groups

23.0 Interview/Focus Groups:

23.1 **Does this study involve Interviews/Focus Groups?** No

If you answer no and hit continue you will go to the next page.

Answer yes or no.

23.2 **Attach copies of any scripts/or questions that will be used to guide the interview focus/groups:**

Attach scripts or questions.

Name	Version
There are no items to display	

23.3 **Idendtify all Staff conducting interviews on page 1 question 1.4 by selecting the correct role.**

23.4 **Is there any specific training or qualifications needed to conduct the interviews/focus groups?**

Describe training and/or qualifications.

View: 24-00 Psychological Testing

24.0 Psychological Testing:

24.1 **Does this study involve Psychological testing?** No

If you answer no and hit continue you will go to the next page.

Answer yes or no.

24.2 **First Please list all Psychological Tests that will be given:**

First please list the test (s)/measures to be used.

24.3 **Attach copies of all psychological test(s)/measures that will be used for this study:**

Second attach copies of all test (s)/measures.

Name	Version
There are no items to display	

24.4 **Is there any necessary training or licenses required of those administering the psychological testing?**

Idendtify all Staff Administering tests on page 1 question 1.4 by selecting the correct role.

Describe any training or licenses required to administer test(s).

View: 25-00 Surveys/Questionnaires

25.0 Surveys/Questionnaires:

25.1 **Does this study involve Surveys/Questionnaires?** Yes

If you answer no and hit continue you will go to the next section.

Answer yes or no.

25.2 **Please attach all questionnaires and/or surveys to be used in this study:**

Attach survey(s)/questionnaire (s).

Name	Version
Harvard Food Frequency Questionnaire | History	0.01
Leisure Time Activity Questionnaire | History	0.01

25.3 **Idendtify all Staff conducting Surveys on page 1 question 1.4 by selecting the correct role.**

View: 26-00 Deception

26.0 Deception:

Deception is a research methodology. When deception is used in research the subject is not told, or is misled, about the true purpose of the research, such as in certain studies of group processes, contextual influences on cognition, etc.

26.1 **Does this study involve the use of deception as a study design method for the research?**
No

If you checked no then hit the continue button and you will be taken to the next page.

Deception is defined as intentionally misleading or withholding information about the nature of the experiment.

26.2 **Describe in detail the nature of the deception and explain why this is necessary for the research:**

Please describe the nature of the deception.

26.3 **State how, when and by whom the research subjects will be debriefed:**

Briefly describe your plan to debrief subjects.

View: 27-00 Additional Documents

27.0 Additional Documents:

27.1 **Are there any additional study documents you wish to attach to this application?**

Name	Version
There are no items to display	

Attach any additional study documents i.e protocols supplied by sponsor.

View: The End

<u>To Finalize this application you must do two things:</u>

1.) As a final step you should click on Hide/Show Errors on the top of this page. If there are any required fields in the Application you have omitted they will show up in red. If you click on each item you will be taken to that page of the application so you can complete the question.

Note: Unless all named Co-investigators have agreed to participate you will not be able to submit your study. Co-Investigators have to press the Co-Investigators agree to participate button. You can send them an email message telling them to do this by pressing Notify Co-Investigators of Need to Agree to Participate. The minute you have selected your Co-Investigators you can press this button it is not advisable to wait until you have completed the application as it may hold up your submission.

When all error messages are gone then...

2.) Click Finish

Please click on the "Finish" button to finalize and exit the Study application. Doing so will **<u>NOT</u>** submit the application for review.

3.) The PI must press the Submit Study button (when they are ready to submit to the IRB)

Please note that a submission may <u>only</u> be forwarded to the IRB by the Principal Investigator. To do this, the Principal Investigator must push "**Submit Study"** in the blue area on the left hand side of the page under **My Activities**. Only the PI will have this button it will not be visible to any other study team members.

You can track the ongoing status of your submission by logging into the study workspace. On the top left hand side of the page in the light blue area there will be a box labeled with the **Current State** of your study.

Please contact the IRB with any questions or concerns. When calling the IRB Office Please direct your questions to the IRB staff named as the "Owner" of your study.

View: CCF Key Personnel Questions View
*** Name of Key Personnel Working on Study:** Praneeta Chodavarapu

Study Role:

Name
Research Support Staff

View: CCF Key Personnel Questions View
*** Name of Key Personnel Working on Study:** Perrie O'Tierney-Ginn

Study Role:

Name
Research Support Staff

View: CCF Key Personnel Questions View
*** Name of Key Personnel Working on Study:** Sylvie Hauguel-de Mouzon

Study Role:

Name
Co-investigator
Interviewer (Survey, Focus Group)
Obtaining Informed Consent

View: CCF Key Personnel Questions View
* **Name of Key Personnel Working on Study:** Shoi Smith

Study Role:

Name
Research Support Staff
Interviewer (Survey, Focus Group)
Obtaining Informed Consent

View: CCF Key Personnel Questions View
* **Name of Key Personnel Working on Study:** Sharon Groh-Wargo, PhD

Study Role:

Name
Co-investigator
Interviewer (Survey, Focus Group)
Obtaining Informed Consent

View: CCF Key Personnel Questions View
* **Name of Key Personnel Working on Study:** Stephen Myers

Study Role:

Name
Research Support Staff

View: CCF Key Personnel Questions View
* **Name of Key Personnel Working on Study:** Larraine Presley

Study Role:

Name
Study Coordinator
Research Support Staff
DRA (only one)
Interviewer (Survey, Focus Group)
Obtaining Informed Consent

View: CCF Key Personnel Questions View
* **Name of Key Personnel Working on Study:** Patrick Catalano

Study Role:

Name
Interviewer (Survey, Focus Group)
Obtaining Informed Consent

View: CRU DSMP Data Collection Simple View
Name: Chart Review, interview, questionnaire
Level of Risk: Minimal and Low Risk Studies
Type: Data Collection
View: CRU DSMP Data Collection Simple View
Name: Anthropomorphic evaluations
Level of Risk: Minimal and Low Risk Studies
Type: Data Collection
View: CRU DSMP Data Collection Simple View
Name: Pregnant Population
Level of Risk: Moderate Risk Studies
Type: Study Population
